# Nucleotide substrate binding characterization in human pancreatic-type ribonucleases

**DOI:** 10.1371/journal.pone.0220037

**Published:** 2019-08-08

**Authors:** Khushboo Bafna, Chitra Narayanan, S. Chakra Chennubhotla, Nicolas Doucet, Pratul K. Agarwal

**Affiliations:** 1 Genome Science and Technology, University of Tennessee, Knoxville, Tennessee, United States of America; 2 INRS-Institut Armand-Frappier, Université du Québec, Laval, Québec, Canada; 3 Department of Computational and Systems Biology, University of Pittsburgh, Pittsburgh, Pennsylvania, United States of America; 4 PROTEO, the Quebec Network for Research on Protein Function, Structure, and Engineering, Université Laval, Québec, Quebec, Canada; 5 Department of Biochemistry & Cellular and Molecular Biology, University of Tennessee, Knoxville, Tennessee, United States of America; King's College London, UNITED KINGDOM

## Abstract

Human genome contains a group of more than a dozen similar genes with diverse biological functions including antiviral, antibacterial and angiogenesis activities. The characterized gene products of this group show significant sequence similarity and a common structural fold associated with binding and cleavage of ribonucleic acid (RNA) substrates. Therefore, these proteins have been categorized as members of human pancreatic-type ribonucleases (hRNases). hRNases differ in cell/tissue localization and display distinct substrate binding preferences and a wide range of ribonucleolytic catalytic efficiencies. Limited information is available about structural and dynamical properties that influence this diversity among these homologous RNases. Here, we use computer simulations to characterize substrate interactions, electrostatics and dynamical properties of hRNases 1–7 associated with binding to two nucleotide substrates (ACAC and AUAU). Results indicate that even with complete conservation of active-site catalytic triad associated with ribonucleolytic activity, these enzymes show significant differences in substrate interactions. Detailed characterization suggests that in addition to binding site electrostatic and van der Waals interactions, dynamics of distal regions may also play a role in binding. Another key insight is that a small difference in temperature of 300 K (used in experimental studies) and 310 K (physiological temperature) shows significant changes in enzyme-substrate interactions.

## Introduction

A set of related human genes encoding for enzymes that cleave ribonucleic acid (RNA) substrates also exhibit diverse biological functions such as angiogenesis, antiviral, antibacterial, and/or cytotoxic activities. Eight of the encoded enzymes (*canonical members*) show conservation of two active-site histidine residues and a lysine associated with the catalytic mechanism of ribonucleolytic cleavage. An additional five catalytically inactive (*non-canonical members*) pseudogenes were also identified in the human genome [1–4]. Collectively, these 13 proteins exhibit 28% sequence identity (40% among the 8 canonical members). All structurally characterized members share a common structural fold while displaying a diverse range of catalytic efficiencies and biological activities [5, 6]. These proteins are referred to as human ribonucleases (hRNases) due to their conserved fold and ribonucleolytic function, which was first discovered and characterized for bovine ribonuclease A (bRNaseA). As enzymes, hRNases catalyze the transphosphorylation and hydrolysis of the phosphodiester bond in single-stranded RNA substrates, although cleavage of double-stranded RNA has also been reported [7, 8]. More broadly, the hRNases and bRNaseA belong to pancreatic-type ribonucleases (RNases) superfamily of proteins with about 1500 members, mostly from mammals.

**Fig 1:**
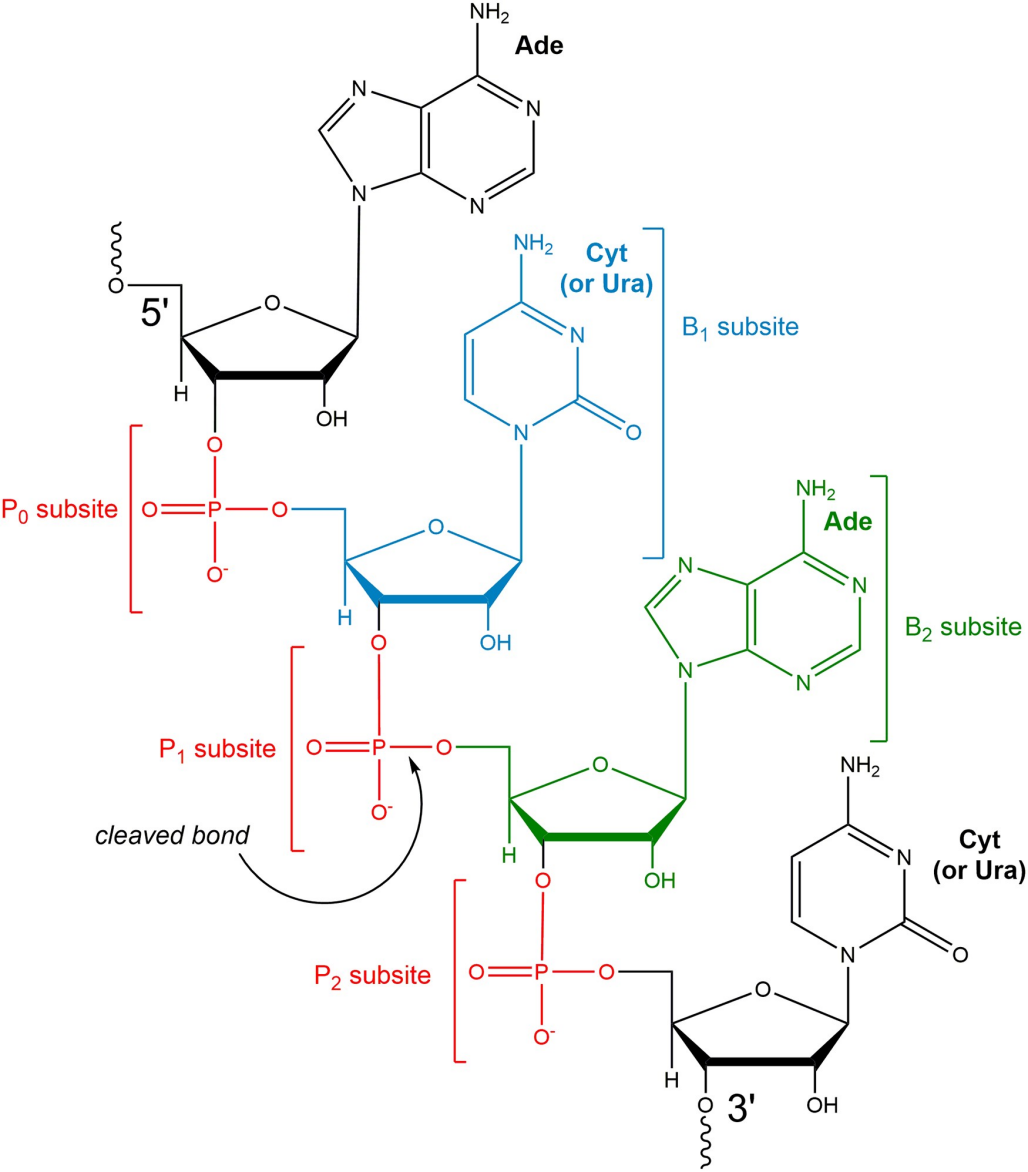
RNase-substrate binding sub-sites. Schematic representation based on the bRNaseA active-site. P_0_, P_1_ and P_2_ represent binding sub-sites for the phosphate groups of the RNA substrate backbone, while B_1_ and B_2_ correspond to nucleotide base sub-sites. The cleaved phosphodiester bond is indicated by an arrow. The nucleotide bases in pockets B_1_ (cytosine drawn, but uracil can also bind) and B_2_ (adenosine) are rendered based on published information about nucleotide preference in bRNaseA; for some hRNases, the information about nucleotide preference is unclear. Figure redrawn based on [7]. The substrates investigated in this study are 5’-ACAC-3’ (shown) and 5’-AUAU-3’. In the present work, simplified nucleotides numbering scheme A_(-2)_C/U_(-1)_A_(1)_C/U_(2)_ is used with negative numbers corresponding to nucleotides away from cleaved phosphodiester bond decreasing in the 5’ direction and positive numbers for nucleotides away from cleaved phosphodiester bond increasing in the 3’direction.

RNase superfamily members bind and catalyze the phosphodiester bond cleavage in a wide variety of nucleotide substrates of different lengths and sequences. Substrate binding and kinetic assays for some hRNases have been performed using a number of substrates with varying nucleotide sequences and lengths, resulting in a wide variety of binding affinities and catalytic efficiencies being reported [9–27]. Structural studies have provided detailed information for binding sites for the phosphate groups (P_0_, P_1_, P_2_), pyrimidine (B_1_) and purine (B_2_) nucleotide bases in the active-site of bovine RNase A, the archetypal member of this superfamily (Fig 1)[7]. The past studies have suggested substrate preferences at the B_1_ pyrimidine binding site for some of the human RNases: hRNase1 showed a preference for cytosine (C) over uridine (U), while hRNases 2, 3, 4 and 6 showed a preference for U over C [6, 9]. In contrast, the B_2_ purine binding site was shown to prefer adenosine (A) in these hRNases [6, 9, 28]. Substrate preferences for the other hRNases remains unclear [29]. While RNase homologs exhibit specific nucleotide sub-site preferences in the context of structurally distinct active-site pockets, reports have illustrated that binding affinities differ over several orders of magnitude between all hRNase members (*K*_*M*_ ranging from about 2 μM to 250 mM and *k*_*cat*_ ranging from 0.003 to 2400 s^-1^; see S1 Table in supporting information for details) [9–27]. As hRNases exhibit distinct substrate specificities and bind a wide range of non-specific RNA sequences with micromolar affinities, the diverging ribonucleolytic preferences and catalytic activities has created challenges in understanding their chemical and designated biological functions at the molecular level. Therefore, considerable interest exists in the characterization of the structure, dynamics and catalytic mechanisms of hRNases, in order to gain a better understanding of their biological functions in the cell.

One of the most intriguing aspects of hRNases is the wide range of catalytic efficiencies associated with ribonucleolytic activities observed for these homologs, which differ by as much as 10^6^-fold [5, 6]. Interestingly, the time-scales associated with their intrinsic dynamics (corresponding to large-scale global conformational exchange) also varies over 5–6 orders of magnitude [5]. The similarity in range of catalytic efficiencies as well as the observed intrinsic dynamics raises an interesting question regarding the relevance of dynamics in the catalytic mechanism [5]. Recently, structural and dynamical characterization of a group of 23 members of the RNase superfamily based on nuclear magnetic resonance (NMR) and computational simulations (of apo proteins) provided unique insights into the conservation of dynamical properties within this enzyme family [30]. Members within sub-families, identified based on phylogenetic classification, showed similarity in structure, and more interestingly, similarity in biological function and intrinsic dynamics. Furthermore, the different sub-families corresponding to branches of evolution associated with gene duplication followed by functional adaptation show largely different dynamics. For a direct comparison of the catalytic efficiencies and dynamics of these hRNases, the use of identical substrates in similar conditions is essential to eliminate effects caused by inhomogeneous nucleotide sequences and/or experimental variations.

In this study, we use computer modeling to investigate the binding and interactions of 8 canonical members of the RNase superfamily (hRNase 1–7 and bovine RNase A) in the presence of two model single-stranded RNA substrates: the tetraribonucleotide sequences ACAC and AUAU. The selection of these model substrates is based on previous experimental data on nucleotide preferences in the B_1_/B_2_ sub-sites as well as structural and dynamical characterization of hRNases from our group [5, 30]. Using microsecond time-scale molecular dynamics (MD) simulations, we characterized the binding stability, hydrogen bonding, van der Waals and electrostatic interactions between the enzymes in complex with the two model substrates. The detailed comparative analysis provides new insights into the structural and dynamical properties that contribute to enzyme-nucleotide substrate interactions in these RNases. The results indicate that while the 8 enzymes display conservation of the catalytic triad and a number of active-site residues involved in substrate interactions and stability, their substrate preference and binding affinities differ significantly. These results further indicate that, in addition to the conserved catalytic triad, other active-site residues play important roles in enzyme-substrate interaction and stability, and therefore, could have important consequences for differences in the biological function between functional and structural enzyme homologs. Somewhat surprisingly, a temperature difference from 300 K to 310 K shows significant differences in substrate stability behavior in these enzymes. This is intriguing because the vast majority of prior experimental enzyme characterization were performed at room temperature (~300 K), while the relevant physiological temperature for these enzymes is closer to the average human (~310 K) and bovine (~312 K) body temperatures. These observations highlight the effect of subtle changes in temperature on the structural and dynamical properties of enzymes and further illustrate the importance of the selection of temperature in experimental and computational studies that are representative of physiological conditions.

## Methods

### Atomic coordinates

Seven human ribonuclease substrate complexes were prepared based on X-ray/NMR structures with the following PDB codes: 2K11 (hRNase1) [31], 1GQV (hRNase2) [32], 1QMT (hRNase3) [33], 1RNF (hRNase4) [34], 1ANG (hRNase5) [35], 2HKY (hRNase7) [36] and 7RSA (bRNaseA) [37], while hRNase6 was crystallized in our group [38]. The sequence alignment for these 8 protein systems is provided in S1 Fig of supporting information. hRNase8 was not investigated in this study as structural information about this enzyme is not yet available. Each of these proteins were investigated complexed with two model substrates AUAU and ACAC, under explicit water conditions based on the procedure described below.

### Computational systems preparation

Computational system preparation and all-atom MD simulations were performed using the AMBER simulation package [39]. For preparation of the nucleotide substrate, the coordinates for the phosphate and ribose sugar backbone were adopted from the DNA ligand (ATAA) in PDB structure 1RCN [40]. The nucleotide bases of the substrate template were modified to create the desired nucleotide (ACAC or AUAU), using AMBER’s *leap* modeling preparation module. Water molecules from the PDB structures were not used for model preparations to avoid any overlaps with modeled substrate. For protein residues with multiple conformations in the PDB files, the coordinates for first conformation were used. AMBER’s *ff14SB* force-field were used for modeling both protein residues and RNA substrates. The protein-ligand complex was neutralized with the addition of chloride (Cl^-^) counterions and solvated by immersing in a rectangular box (SPC/E water model) [41] with periodic boundary conditions. The prepared system was equilibrated using the protocols developed in our group and described previously [42, 43]. These protocols were effective in fully resolving the steric clashes caused by the introduction of modeled substrate in the active-site (see supporting information and S2 Fig for more details). AMBER’s *pmemd* simulation engine was used for equilibration and production runs, with PME-method for long range electrostatics [44]. All hydrogen bonds were constrained using SHAKE [45].

### Histidine protonation states

There are two conserved histidine residues in the active-site of all RNases that participate in the phosphodiester bond cleavage mechanism. The protonation states for these residues were determined based on the past pH profiles for bRNaseA as well as structural interaction with modeled substrate and other residues [46, 47]; the two histidine residues His12 and His119 (bRNaseA numbering), were modeled in single (proton on N_δ1_) and double protonated states respectively. All the other histidine residues were modeled in single protonated state for all 8 RNases.

### MD simulations

Equilibration runs were performed under constant volume and temperature (NVT) conditions with a thermostat to bring the system to target temperature of 300 K or 310 K for production run (see below). Production runs of 0.5 μs (microsecond) were performed under constant volume and energy (NVE) simulation conditions using a 2 femtoseconds (fs) time-step. Production runs under NVE ensemble were performed as this ensemble offers better computational stability and performance for longer MD simulations [48]. All simulations were performed using NVIDIA graphics cards with CUDA-enabled version of AMBER’s *pmemd* MD simulation engine. The default precision in the *pmemd*.*cuda_SPFP* binary was used from AMBER v14 package.

**Fig 2:**
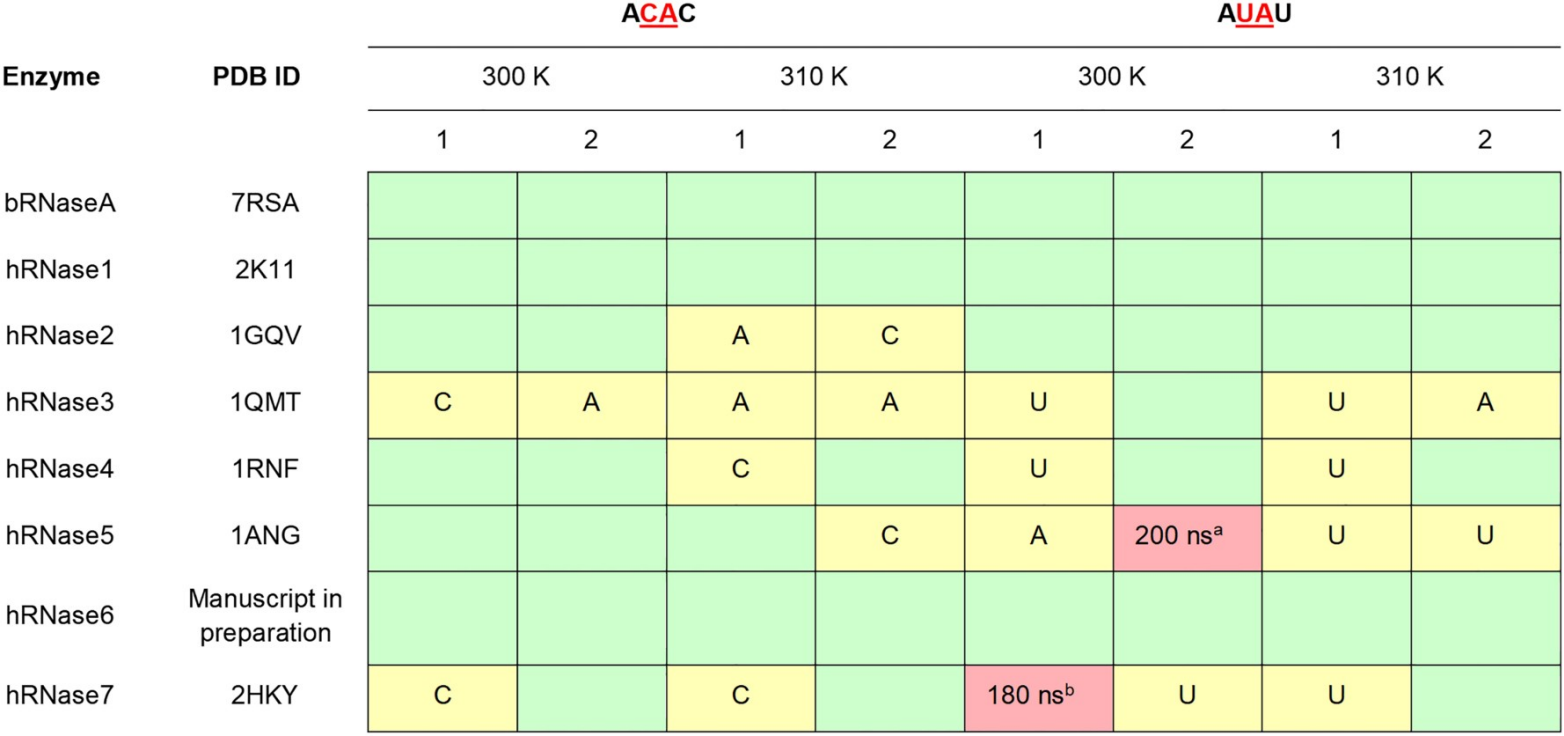
Stability of nucleotide substrates ACAC and AUAU in the active-site of RNases. Stable substrates in the active-site for entire 0.5 μs (500 ns) are colored as green boxes; cases where one nucleotide is ejected are colored yellow and the stable nucleotide base is labeled (A–adenine, U–uracil, C–cytosine); and cases where both nucleotides are ejected are indicated as red boxes (time in nanoseconds when the substrate is fully ejected is specified). Note that only the two central nucleotides were considered for this analysis (in red, underlined). ^a^ = additional (3^rd^) trajectory indicates that only central U remained in active-site, ^b^ = additional (3^rd^) trajectory indicates that the substrate was ejected from active-site at 370 ns.

Two independent sets of simulations were performed at 300 K and 310 K (no thermostat was applied during production runs). Furthermore, to ensure the reproducibility of the results from this study, two independent trajectories were computed at each temperature (labeled as 300 K 1, 300 K 2, 310 K 1, and 310 K 2 in the remaining text and Fig 2). For the set 2, after equilibration the system was minimized (100,000 steps) followed by MD (1000 ps) to slowly readjust the temperature before the production runs were performed. At each temperature, for each of the unique RNase-substrate complex, 2,000 structures (1,000 from each trajectory, 500 picoseconds apart) were stored and analyzed for structural, electrostatic and dynamical analysis, as described below. A subset of stored structures was used for hRNase5 (1,400 structures) and hRNase7 (1,360 structures) analysis as the substrates were ejected mid-way during the simulations; therefore, only the structures where the substrate stays bound in the sub-sites were used for analysis. Specifically, only the first 400 structures from hRNase5-AUAU 300 K 2 trajectory and first 360 structures from hRNase7-AUAU 300 K 1 trajectory were used for structural (H-bonding) and interaction energy analyses as described below. For the two systems where the substrate was ejected (hRNase5-AUAU at 300 K and hRNase7-AUAU at 300 K), addition MD simulations were performed to ensure the reproducibility of the behavior. To generate the starting coordinate for this third set of trajectory for these two systems, the equilibrated structures were heated for 1000 ps to 305 K (for simulation set for 300 K) and 315 K (for simulation set for 310 K) to generate conformational diversity. The ending structures were minimized for 100,000 steps and heated to final temperature before production runs were performed similar to the other two sets.

### Structural analysis using H-bonding patterns

H-bonding interactions between the substrate and RNases were analyzed using *ptraj*, with the following criteria: less than 3.3 Å for donor to acceptor heavy atom distance at an angle between 135° and 180° for the donor-hydrogen-acceptor. The percentage occupancies of H-bonds, defined as fraction (percentage) of total number of structures (as described in last paragraph) where the interaction meets the H-bonding criterion mentioned above, were also calculated.

### Enzyme-substrate interaction

The energy for the enzyme-substrate interactions (*E*_*enz-subs*_) were calculated as a sum of electrostatic (*E*_*el*_) and van der Waals energy (*E*_*vdw*_) between atom pairs, based on an approach developed in our group [42, 49]. Details of the methodology are provided in supporting information. All enzyme and substrate (ACAC/AUAU) atom pairs were included in the calculations and resulting interaction energies were summed for each residue-nucleotide pair. For each system, the reported interaction energies are an average over total number of structures (as described above). This method has successfully been used to predict the experimentally obtained binding preference/affinities of substrates for a number of proteins including human cyclophilin A, xylanases, and maltose binding proteins similar to the substrate preference of hRNases described in this study [42, 49, 50].

### Electrostatic surface calculations

The enzyme electrostatic surface was computed using the Adaptive Poisson-Boltzmann Solver (APBS) software [51]. APBS requires a protein conformation. The ideal case of using averaged enzyme structure for the entire MD simulation was not feasible as the averaged structure shows artificial bonds and angles, particularly to the sampling of different conformations over the 0.5 μs simulations. Therefore, a conformation from the MD ensemble closest to the averaged structure (with least RMSD to the average) was used to generate the electrostatic surface.

### RMSF_10_ calculations

For dynamical analysis, backbone (C_α_) and all-atom flexibility of simulation trajectories was determined from the root mean square fluctuation (RMSF), computed by aggregating the magnitude of displacement eigenmodes computed using the quasi-harmonic analysis (QHA) in the *ptraj* analysis module in AMBER. As described previously [52, 53], only the top 10 QHA modes (RMSF_10_) were used in the analysis to focus on the principal dynamics or long time-scale functionally relevant fluctuations in the proteins. For comparison, the results for apo systems were obtained from our previous study [30].

## Results

### Stability of the substrate-bound RNase complexes

Fig 2 summarizes the interactions of hRNases 1–7 in complex with model tetranucleotide substrates ACAC and AUAU. Starting from the enzyme conformation with substrate-bound in the active-site, 0.5 μs MD simulations were used to characterize the stability of interactions between the enzyme and nucleotide substrates. Note, the starting coordinates for the substrate phosphate and ribose sugar backbone were adopted from a bRNaseA-substrate complex published previously (PDB code 1RCN), and the nucleotide bases were added computationally (see Methods section for more details). Two independent MD simulation trajectories for each complex were performed, labeled 1 and 2 in Fig 2, to ensure reproducibility of the results reported. These trajectories in PDB format are available in supporting information. The characterization of enzyme-substrate complex stability is based on interaction analysis between the two central nucleotides and enzyme residues. Only the two central nucleotides were considered for stability analysis as these nucleotides are adjacent to the phosphodiester bond cleaved by RNases and have well defined interactions with active-site residues forming the P_1_/B_1_ and P_2_/B_2_ binding sites (see Fig 1). The other/terminal two nucleotides in the model substrates do not have well defined interactions and show significantly lower stability over the course of the MD simulations. In Fig 2, simulation trajectories where the two central nucleotides show stable binding in their respective sub-sites (for entire 0.5 μs) are represented with a green box, while trajectories where one of the two central nucleotides is ejected from the sub-site are shown using a yellow box. The stable nucleotide in the binding sub-site is identified in the yellow boxes of Table 1. The cases where both the central nucleotides lose all contacts with the sub-site residues and are considered to be ejected out of the binding pocket are marked in red boxes. Ejection out of the binding pocket is defined as no direct hydrogen bond or van der Waals contact between the enzyme residues and the two central nucleotides (any water or ion-mediated indirect interactions were excluded from this analysis).

**Table 1:** H-bonding properties of bovine and human pancreatic-type RNases at 300 K. The H-bonds that meet the criteria of 3.3 Å, angle between 135–180° and more than 45% occupancy are listed. Results are based on analysis of both the alternate MD trajectories for each system (see Methods for details).

	ACAC			AUAU		
	Substrate	Residue	bond length (% occupancy)	Substrate	Residue	bond length (% occupancy)
**bRNaseA**	C_(-1)_ (N_3_)C_(-1)_ (O_2_)A_(1)_ (N_1_)C_(-1)_ (O_2'_)A_(1)_ (N_6_)	Thr45 (O_ϒ1_)Thr45 (N)Asn71 (N_δ2_)Phe120 (O)Asn71 (O_δ1_)	2.87 (95.7%)2.87 (88.5%)3.01 (65.0%)2.74 (47.2%)3.00 (46.4%)	U_(-1)_ (O_2_)U_(-1)_ (N_3_)A_(1)_ (O_P2_)A_(1)_ (N_6_)A_(1)_ (N_1_)A_(1)_ (O_P1_)	Thr45 (N)Thr45 (O_ϒ1_)Gln11 (N_ε2_)Asn71 (O_δ1_)Asn71 (N_δ2_)Phe120 (N)	2.88 (95.4%)2.97 (85.8%)2.84 (73.2%)2.95 (69.2%)3.02 (68.8%)2.93 (58.6%)
**hRNase1**	C_(-1)_ (N_3_)C_(-1)_ (O_2_)C_(-1)_ (O_2'_)	Thr45 (O_ϒ1_)Thr45 (N)Phe120 (O)	2.89 (93.6%)2.89 (72.2%)2.75 (49.2%)	U_(-1)_ (N_3_)A_(1)_ (N1)U_(-1)_ (O_2'_)A_(1)_ (N6)	Thr45 (O_ϒ1_)Asn71 (N_δ2_)Phe120 (O)Asn71 (O_δ1_)	2.91 (98.4%)2.98 (94.2%)2.73 (87.0%)2.98 (85.6%)
**hRNase2**	C_(-1)_ (N_3_)A_(1)_ (O_P2_)	Thr43 (O_ϒ1_)Gln15 (N_ε2_)	2.88 (79.4%)2.87 (65.6%)	U_(-1)_ (N_3_)A_(1)_ (O_P2_)U_(-1)_ (O_4_)	Thr43 (O_ϒ1_)Gln15 (N_ε2_)Ile134 (N)	2.95 (66.1%)2.88 (55.8%)2.98 (47.7%)
**hRNase3**	A_(1)_ (N_6_)A_(1)_ (N_1_)	Asn71 (O_δ1_)Asn71 (N_δ2_)	2.95 (51.6%)3.01 (50.8%)	A_(1)_ (N_6_)A_(1)_ (N_1_)5’A_(-2)_ (N_6_)	Asn71 (O_δ1_)Asn71 (N_δ2_)Thr133 (O)	2.96 (67.4%)3.01 (67.0%)2.89 (60.0%)
**hRNase4**	A_(1)_ (O_P1_)A_(1)_ (O_P1_)C_(-1)_ (N_4_)A_(1)_ (N_1_)A_(1)_ (N_6_)A_(1)_ (O_5'_)	Phe118 (N)His117 (N_δ1_)Thr45 (O_ϒ1_)Asn71 (N_δ2_)Asn71 (O_δ1_)His117 (N_δ1_)	2.91 (92.9%)2.80 (66.3%)2.91 (63.1%)3.00 (61.0%)2.94 (59.9%)2.96 (54.2%)	U_(-1)_ (N3)U_(-1)_ (O_2_)A_(1)_ (O_P1_)	Thr45 (O_ϒ1_)Thr45 (N)Phe118 (N)	2.95 (93.8%)2.93 (86.2%)2.89 (59.0%)
**hRNase5**	C_(-1)_ (N_4_)A_(1)_ (O_P1_)	Thr44 (O_ϒ1_)His114 (N_δ1_)	2.95 (63.0%)2.77 (58.6%)			
**hRNase6**	A_(1)_ (O_P1_)A_(1)_ (N_1_)A_(1)_ (N_6_)A_(1)_ (O_P1_)A_(1)_ (N_6_)C_(-1)_ (O_2'_)	Gln15 (N_ε2_)Asn69 (N_δ2_)Asn69 (O_δ1_)Trp11 (N_ε1_)Asn65 (O_δ1_)Leu124 (N)	2.92 (85.8%)3.02 (84.8%)2.98 (84.4%)2.91 (79.7%)2.98 (50.6%)3.00 (46.9%)	A_(1)_ (N_6_)A_(1)_ (N_1_)3’U_(2)_ (O_4_)A_(1)_ (N_6_)A_(1)_ (O_P2_)	Asn69 (O_δ1_)Asn69 (N_δ2_)Arg67 (N_η1_)Asn65 (O_δ1_)His123 (N_ε2_)	2.93 (96.2%)3.05 (88.0%)2.93 (83.1%)2.94 (70.7%)2.94 (52.0%)
**hRNase7**	C_(-1)_ (N_3_)	Thr43 (O_ϒ1_)	2.85 (53.2%)	U_(-1)_ (O_2'_)5’A_(-2)_ (N_6_)	Leu125 (O)Thr43 (O_ϒ1_)	2.67 (70.51%)2.95 (48.01%)

The interaction analysis shows that the majority of RNases show stable nucleotide binding throughout the simulations for the two alternate temperatures at 300 K and 310 K, reproduced in both the alternative MD trajectories. Notable exceptions include hRNase5 and hRNase7, where the substrate AUAU is completely ejected out of the active-site. In the case of hRNase3, the enzyme displays interactions with only one of the two central nucleotides, while the second central nucleotide is ejected out of the binding pocket (Fig 2). There are marked differences associated with the two temperatures as well as two independent trajectories at each temperature, but overall the results are qualitatively similar. Note that these are human proteins and 310 K is close to the physiological temperature, while a number of experimental enzyme kinetics parameters are reported for these systems at temperature close to 300 K [9–13, 22, 24–26, 54]. For hRNase5 and hRNase7, additional data was collected to confirm the observations of the ejection of substrate from the active-site. The third MD set (data only collected for these two systems) indicated that for hRNase7-AUAU at 300 K, the behavior was reproduced, as the substrate was completely ejected from the active-site at 370 ns. For hRNase5-AUAU the behavior was partially reproduced, as 3 out of the 4 nucleotides broke all contacts, with only central U maintaining contact with the protein.

**Fig 3:**
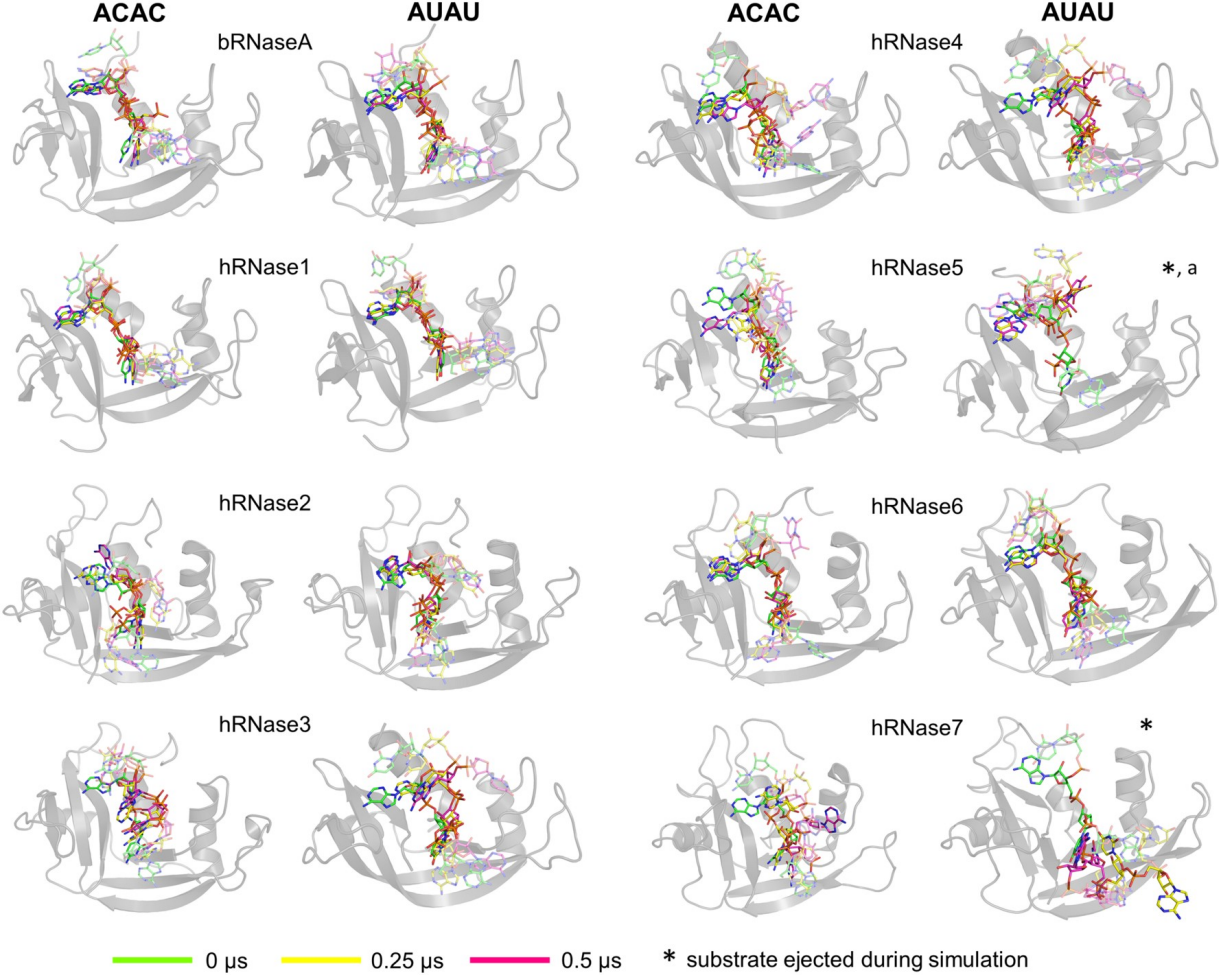
Substrate behavior over the course of 0.5 μs MD simulations. The enzymes are shown as gray cartoons and the 3 orientations of the substrate at start (0 μs, green), mid-way (0.25 μs, yellow) and end (0.5 μs, magenta) of the simulations are shown as sticks. The results for the two substrates ACAC and AUAU are shown separately. The central two nucleotides of the substrate that interact with two binding sub-sites (B_1_/P_1_ or B_2_/P_2_) are shown in dark colors, while the two terminal nucleotides (one on each side) are depicted with faded color. The results depicted are from the 300 K simulation trajectories and represent the MD simulations set 1. The results from trajectory 2 at 300 K are qualitatively similar (Fig 2). * indicates simulations where the substrate is ejected and leaves the active-site; the end and mid-way frames for these cases are adjusted accordingly. ^a^ indicates that the substrate was stable in depicted trajectory (300 K 1); however, it was ejected out from the pocket in second trajectory (300 K 2). Results from 310 K simulations are shown in S3 Fig.

Characterization of the relative substrate positions and interactions with enzymes over the course of MD simulations revealed significant variations among the different RNases. The results are depicted in Fig 3 for simulations at 300 K and S3 Fig for simulations at 310 K (the trajectory labeled 1 in Fig 2 is shown). These figures depict three substrate conformations (relative positions); at the start (0 μs, green sticks for carbon), half-way (0.25 μs, yellow sticks for carbon) and endpoint (0.5 μs, magenta sticks for carbon) of the simulations. In cases where the substrate is completely ejected from the active-site, the mid-way and ending frames have been adjusted according to the time of ejection (listed in Fig 2).

In the cases of bRNaseA, hRNase1, and hRNase6, both model substrates (ACAC and AUAU) stay bound in the active-site for the entire 0.5 μs simulations (300 K as well as 310 K), close to the original orientation, suggesting equal stability of these substrates in these enzymes. Note that the central nucleotides remain close to their initial orientation in the active-site (in P_1_/B_1_ and P_2_/B_2_ sites), while the two peripheral nucleotides show considerable variation from their initial orientation. In the case of hRNase2, both substrates stay in their initial orientation at 300 K, with the central adenosine base showing minor movement out of the B_2_ pocket while phosphate stays bound in the P_2_ pocket. For simulations at 310 K, substrate ACAC is partially displaced from its initial orientation, with the central cytosine in trajectory 1 (adenine in trajectory 2) moving completely out of the P_1_/B_1_ (P_2_/B_2_) pockets (S3 Fig). In the case of hRNase4, substrate ACAC at 300 K stays localized in the active-site while at 310 K adenine leaves the binding pocket; for substrate AUAU at both temperature values adenosine moves out of its binding pocket. In hRNase5, the substrates show considerable movements in simulations at 300 K, particularly in the case of AUAU, where the substrate completely leaves the active-site in one of the MD trajectories (labeled as trajectory 2 in Fig 2). For hRNase3 and hRNase7, at least one of the nucleotide binding to B_1_ or B_2_ site (for both ACAC and AUAU) show significant movement away from the binding pocket over the course of MD simulations at both temperatures investigated.

### Structural interactions

To quantitatively characterize the substrate interactions with each of the 8 proteins in this study, hydrogen bonding interactions were calculated for the ensemble of conformations sampled during the MD simulations. The average distance between heavy atoms of the enzyme and substrate nucleotide, and percentage occupancy of the hydrogen bonding interactions over the course of aggregate 1 μs MD sampling (by combining two simulations labeled as 1 and 2 in Fig 2) were calculated; the results are summarized in Table 1 for 300 K (and S2 Table in supporting information for 310 K) for the two substrates. The results indicate that the conserved catalytic triad (His12, Lys41 and His119, bRNaseA numbering) does not form stable hydrogen bonds, and any definite patterns in the H-bonding interactions between these residues (across all 8 enzymes investigated) and substrates were not observed. Among the non-catalytic active-site residues, the equivalent of bRNaseA residues Thr45 and Asn71 showed most stable H-bond interactions with the substrates in several human RNases. These residues were previously shown to be part of the B_1_ and B_2_ binding sites, respectively [55]. Unsurprisingly, Thr45 and Asn71 are fully conserved in all 8 RNases investigated in this study, possibly illustrating their role in active-site substrate stabilization.

**Fig 4:**
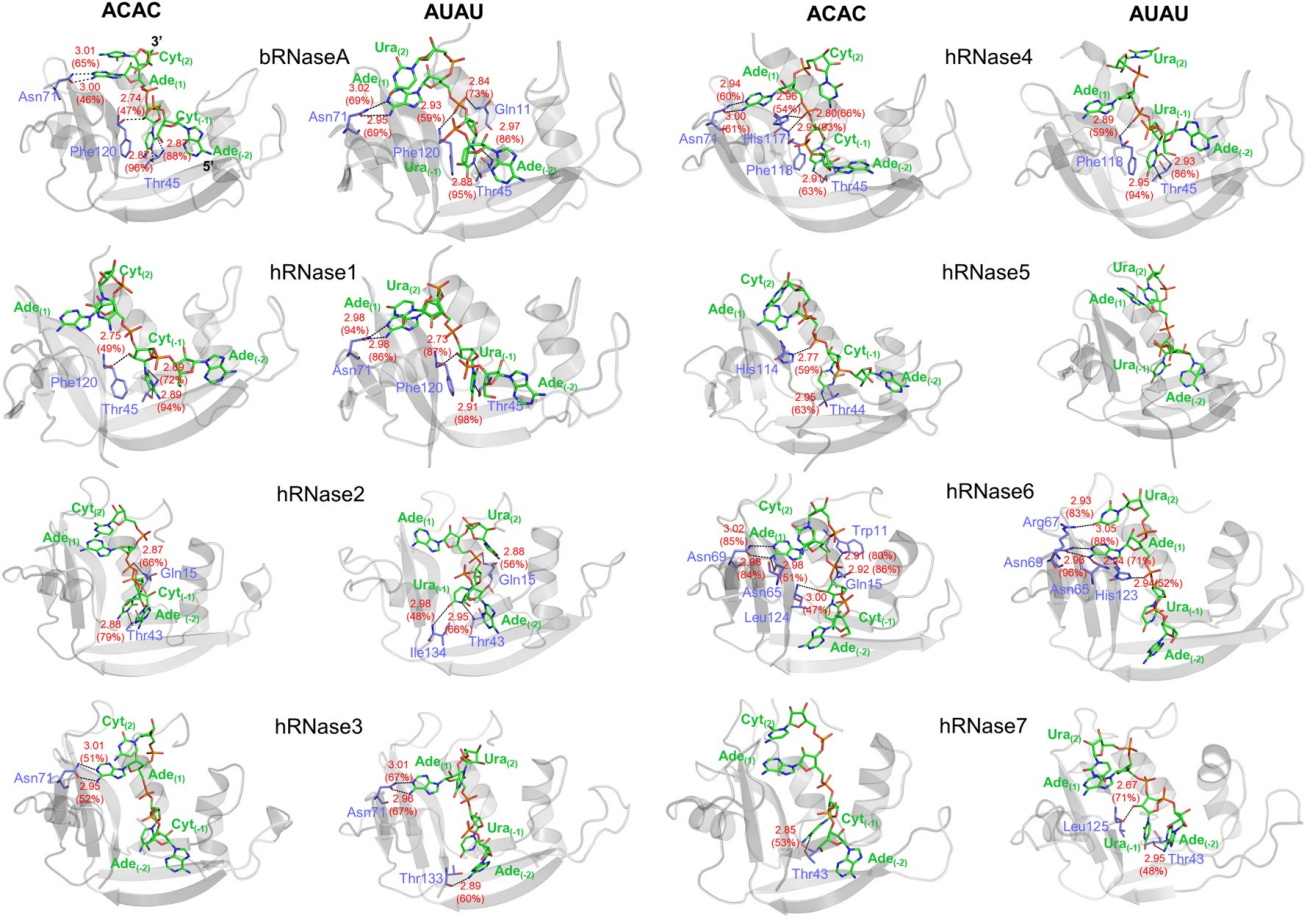
Hydrogen-bonding interactions between RNases and model substrates ACAC and AUAU at 300 K. H-bonds (with >45% occupancy) are indicated with black dotted line and averaged bond length (Å) and percentage occupancy are shown in red colored text. Enzyme and nucleotide atoms participating in the H-bonds are labeled, with enzyme residues shown in blue and substrate nucleotides shown in green for carbon atoms, blue for nitrogen, red for oxygen, and orange for phosphorus. Enzyme residue numbers correspond to enzyme sequence, while substrate nucleotides are numbered as A_(-2)_C/U_(-1)_A_(1)_C/U_(2)_, as described in the legend of Fig 1. Results from 310 K simulations are shown in S4 Fig. hRNase5-AUAU did not show any H-bonds meeting the criteria and is only shown for comparison. Note that the substrates orientation (marked by 3’ and 5’ for bRNaseA) appear opposite to the depiction in Fig 1, as enzymes are shown from view used commonly in literature. In this figure, the percentage occupancies have been rounded off to the nearest whole number.

For enzymes bRNaseA, hRNase1, hRNase4 and hRNase6, strong H-bonding interactions are observed with both substrates at 300 K (Fig 4 and Table 1). In this study, strong H-bonds are defined as H-bonds with bond length less than 3 Å between the heavy atoms; and H-bond occupancies greater than 80% is considered significant. hRNase2 forms at least one H-bond interaction with relatively higher occupancy (79.4%) with substrate ACAC while it forms H-bonds with AUAU that have lower occupancies. On the other hand, hRNase3 and hRNase7 form H-bonds with both substrates that have lower occupancies (≤ 70%). It was also observed that hRNase5 shows no H-bonding interactions with the substrate AUAU. This might be due to the complete ejection of substrate from the binding site of hRNase5 after ~200 ns (0.2 μs) of simulation (Fig 2).

A number of differences were observed when comparing the enzyme-substrate H-bonding interactions at experimental (300 K) and physiological (310 K, see S4 Fig and S2 Table) temperatures with the most obvious difference in H-bonding pattern appears in hRNase3, hRNase5 and hRNase7. Interestingly, hRNases 2 and 3 show lower occupancies with both ligands relative to bRNaseA at both temperatures. For hRNase2, strong H-bonding interactions with substrate ACAC that were observed at 300 K simulations exhibit lower occupancies at 310 K. Simulations indicated that hRNase3 does not form any H-bonds with ACAC at 310 K. A comparison of the interactions at 310 K suggest significantly more interactions for hRNases 3 and 4 with the AUAU ligand. Note when compared to lower temperature, hRNase3 forms H-bonding interactions with AUAU that have lower occupancies. On the other hand, it is observed that hRNase7 does not show any H-bonding interactions with the substrate AUAU at 310 K, although stronger H-bonds are observed with ACAC at 310 K, suggesting a potential preference for cytidine (C) in the B_1_ sub-site. hRNase5, which did not show any H-bonding interactions with substrate AUAU at 300 K, exhibits H-bonds with substrate AUAU at 310 K through the conserved Asn43 and Thr44 residues. The time evolution behavior of H-bonds for various complexes is shown in S5–S12 Figs in supporting information.

Overall, results suggest that even though all the 8 enzymes have conserved catalytic triad and several other conserved residues in the active-site [30], the model substrates show significant differences in their interactions with the enzymes, with some of them showing weak interactions and/or lower occupancies. An interesting observation is that even for substrates which stay bound to the enzymes, the conserved catalytic triad residues do not exhibit strong H-bonding interactions, while other residues show stronger interactions, including residues reported to be involved in substrate recognition and stabilization. This suggests that while other conserved residues may hold the substrate in place, the catalytic triad shows a large degree of flexibility which is possibly required for the catalytical cleavage step. This behavior is different than other enzyme systems where it is shown that the catalytic active-site residues are fairly rigid and make strong interactions with the substrates [56–58]. Note that the structural analysis based on H-bonds does not provide information about complete set of interactions between substrate and hRNase. It helps in identification of the most important direct hydrophilic interactions; however, long range electrostatic and hydrophobic interactions are not captured by H-bonding analysis. In RNases particularly the strongly charged phosphate backbone and the hydrophobic interactions of the nucleotide base in the binding pocket provide important contributions. Therefore, a detailed and quantitative analysis based on full electrostatic and van der Waals interaction energy was also performed as described below.

### Electrostatic interactions

**Fig 5:**
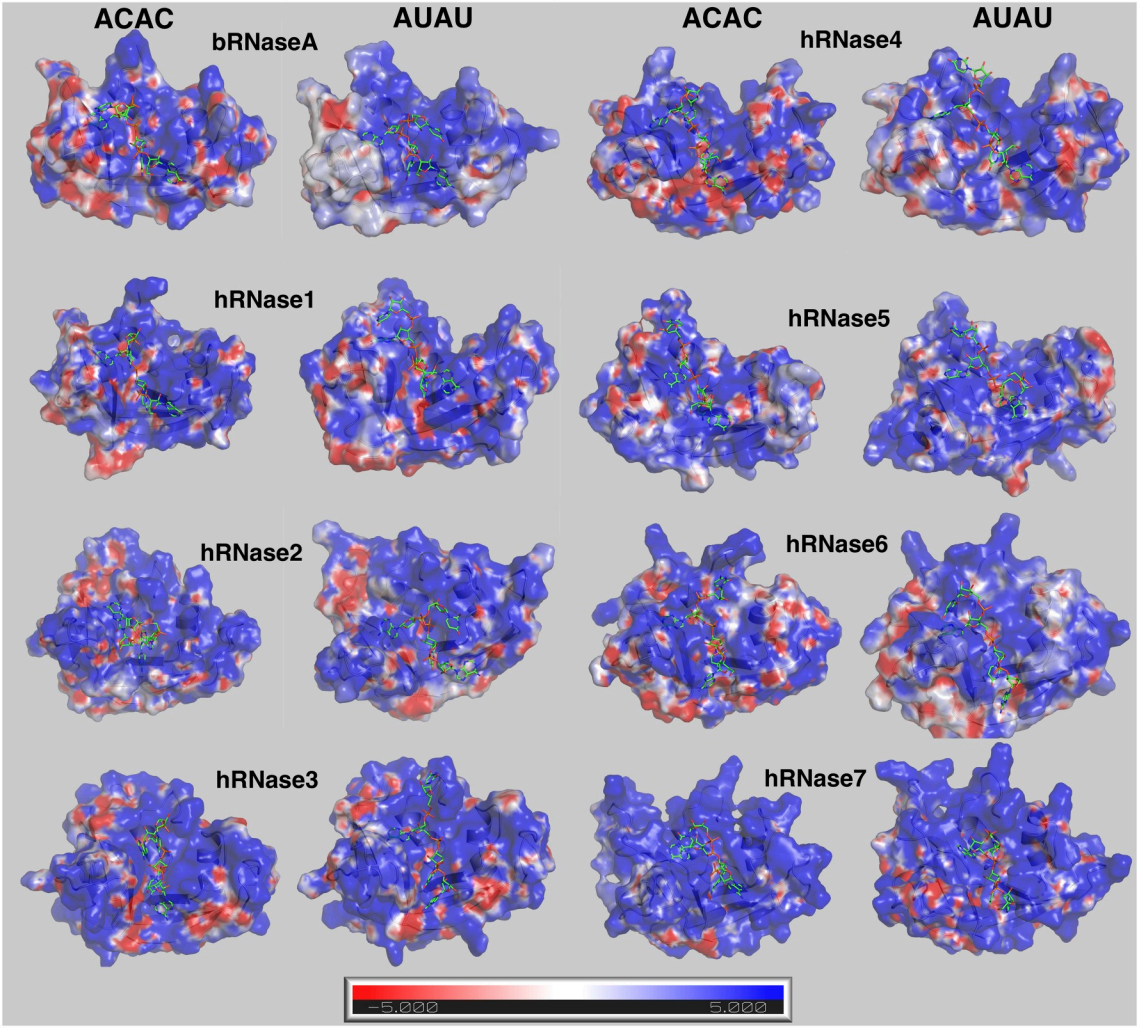
Electrostatic surface of RNases with the two model substrates at 300 K. Enzyme conformation from the MD ensemble closest to the averaged structure for the entire trajectory (conformation with smallest RMSD with averaged structure) was used to calculate the representative electrostatic potential (+5kT/e in blue and –5kT/e per electron in red). Results obtained from simulations at 300 K are shown here; results from the 310 K simulations are shown in S13 Fig.

ssRNA substrates are highly negatively charged molecules, particularly due to the presence of a phosphate backbone. It has been documented that long-range electrostatic interactions play an important role in the binding of negatively-charged RNA substrates to the highly cationic surface of this RNase superfamily [59, 60]. For characterizing and comparing the charge distribution on the surface of these homologs, the averaged electrostatic surface for each enzyme over the course of MD trajectories was calculated (Fig 5 for results obtained from simulations at 300 K and S13 Fig for results from simulations at 310 K). hRNases have been reported to bind to similar substrates and share the same overall shape, but the electrostatic surface shows variations due to the difference in protein sequence. In addition, the charge distribution also showed differences based on the type of bound substrate, possibly indicating changes in orientation of enzyme residues on the surface due to the nature of the substrate. The most prominent feature is that all RNases in this study have highly positively charged surfaces (blue regions in Fig 5). This is not surprising as the RNA substrates are negatively charged, and the positively charged surface is expected to guide the substrate into the active-site pocket. An interesting observation is that the electrostatic surfaces for human enzymes are similar when bound to the two substrates ACAC and AUAU, but different in the case of bRNaseA. Note that bRNaseA is catalytically the most efficient enzyme (for ribonucleolytic cleavage activity) of 8 enzymes investigated in this study. Further studies could investigate the correlation between these enzyme to adapt conformations for optimal electrostatic based on bound substrate and efficiency of ribonucleolytic cleavage.

### Quantitative characterization of interaction energies

**Fig 6:**
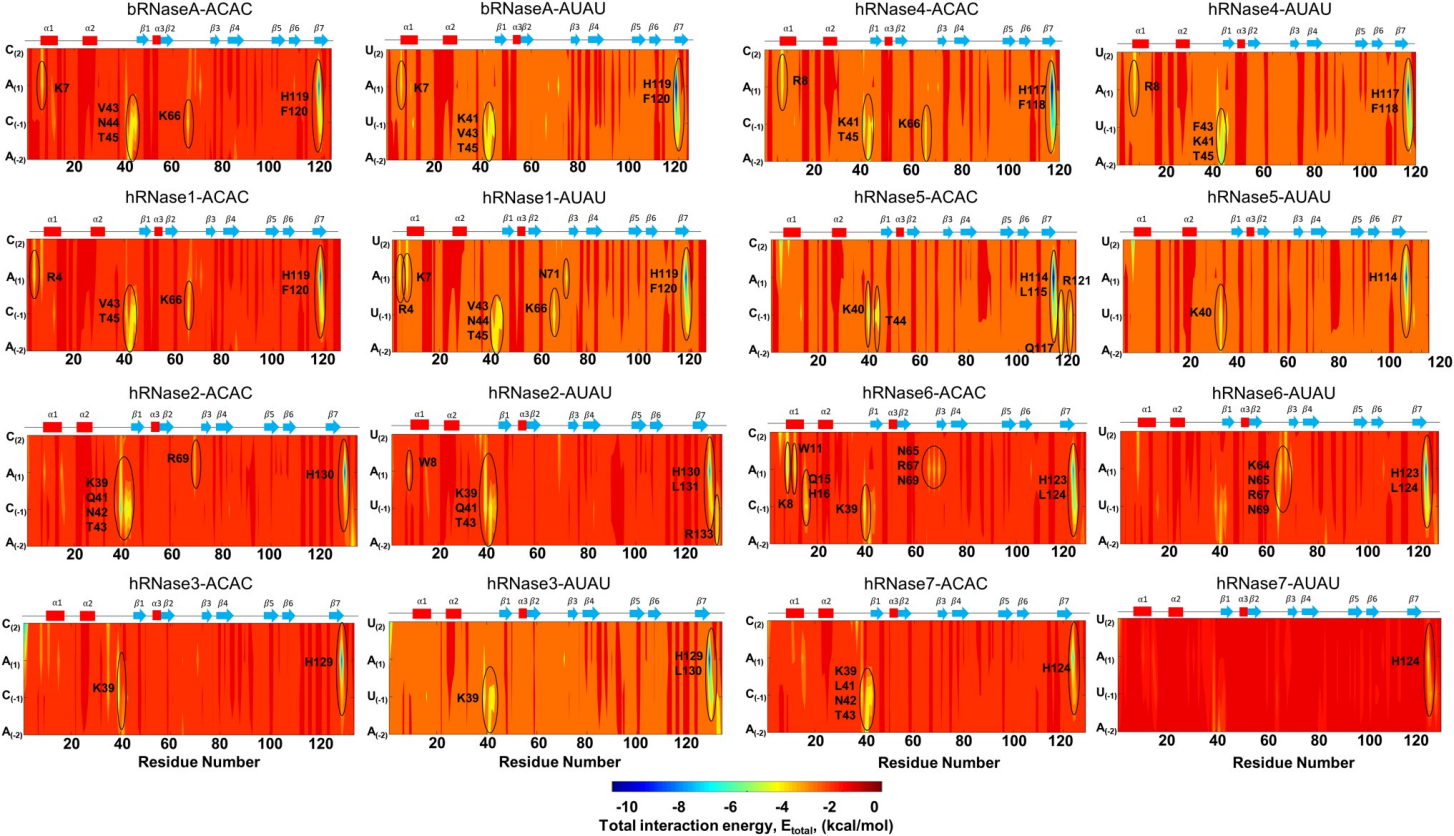
Enzyme-substrate interaction energy at 300 K. Total interaction energy was computed as a summation of electrostatic and van der Waals interaction energy between atom pairs of enzyme residue and substrate nucleotide (ACAC and AUAU, respectively). The areas of largest negative energy (blue) represent favorable interactions, while positive energy (red) represents less favorable or unfavorable interactions. For ease of comparison, sequence of the model substrate has been adjusted to match the orientation depicted in Figs 3 and 4 (CACA and UAUA). Enzyme residues showing total interaction energy (*E*_*total*_, Table 2) contributions < -3 kcal/mol were considered significant and are marked with ellipses. Results depicted are obtained from simulations at 300 K; results from 310 K simulations are shown in S14 Fig.

Fig 6 depicts an overview of the interaction energy between the enzyme and the model substrates, based on the 300 K simulations. The results for 310 K are shown in S14 Fig. The total interaction energy was computed as a sum of van der Waals and electrostatic interaction energies between each enzyme residue and each nucleotide of the substrate molecules. S3 and S4 Tables in supporting information provide the quantitative values, using a cut-off value of < -3 kcal/mol per residue-nucleotide pair being considered as significant. Overall, these results from this analysis indicate that both model substrates show favorable interactions (blue to yellow regions in the figure panels) with the enzyme. Note that H-bonding based structural analysis presented above, describes only a sub-set of key interactions but interaction energy analysis provides a complete picture.

More specifically, favorable interactions with the catalytic His119 (bRNaseA numbering) were observed for both the substrates with all RNases. Most RNases also showed favorable interactions with Thr45 (bRNaseA numbering) in the β1 strand, a residue shown to bind to the pyrimidine nucleotide [28]. bRNaseA residue Lys7 further showed favorable interactions with both the substrates and Lys66 (from loop L4) with substrate ACAC. Similar interactions were observed for hRNase1, which showed additional favorable interactions by Asn71 and Lys7 with AUAU. In case of hRNase2 residues Arg133 and Trp8 show additional interactions in case of substrate AUAU; the latter residue is previously reported to interact with the ribose group [28]. In contrast to hRNase2 where a significant number of interactions are present in the region 39–43, hRNase3 showed significantly fewer interactions with the two substrates. The interaction energy profile of hRNase4 was similar to that observed for bRNaseA for the two substrates. The C-terminal region of hRNase5 displayed favorable interactions with the nucleotide substrates in addition to the catalytic Lys40, however, significantly fewer interactions of hRNase5 were observed with AUAU. In contrast to other RNases, hRNase6 showed favorable interactions with several residues of loop L4, suggesting a potential effect on the dynamical properties of this loop upon substrate binding. hRNase7 showed very few favorable interactions localized to the catalytic His124 with substrate AUAU while substrate ACAC showed additional interactions with the Lys39 and residues of β1 strand. Similar results were observed for all RNases at 310 K except hRNase5 and hRNase7, which showed more favorable interactions with substrate AUAU. See text in supporting information for more details of substrate interactions with each RNase.

A comparison of the total van der Waals (*E*_*vdw*_) and electrostatic (*E*_*el*_) energies summed up for the enzyme-substrate complexes provide a number of interesting observations (Table 2). Even though the nucleotide substrates are highly charged molecules (particularly the backbone phosphate), the largest contribution to the favorable interactions comes from van der Waals interactions, indicating that the active-site residues have preferential interactions with the bases. These quantitative estimates provide additional insights, indicating that bRNaseA possibly has a slight preference for substrate AUAU relative to ACAC, as *E*_*total*_ is lower by 3–5 kcal/mol. In the case of human enzymes, hRNase1 has a better interaction with ACAC at higher temperature (*E*_*total*_ is lower by ~ 9 kcal/mol); hRNase4, RNase5, RNase6 and RNase7 show a preference for ACAC substrate at 300 K, with *E*_*total*_ lower more than 10 kcal/mol; hRNase3 prefers AUAU at 310 K (*E*_*total*_ is lower by almost 9 kcal/mol); while hRNase2 has similar interactions with both substrates. An interesting observation is that hRNase5 and hRNase7, particularly for substrate AUAU, show very weak interactions compared to all the other enzymes in this study. The reason appears to be weak (and even repulsive for hRNase7) electrostatic interactions seen for the AUAU model substrate at 300 K. These quantitative estimates are consistent with the observations that the substrate is ejected midway during the simulations (note the energies were calculated, and averaged, only for conformations where substrates were bound to the enzymes). In the cases of hRNase5 and hRNase7, this observation possibly indicates that active-site residues other than the conserved catalytic triad may not be optimal for the enzyme-substrate interactions (see Fig 6 and S4 Fig, and discussion above). However, as indicated by the H-bond analysis with bound substrate, the non-catalytic residues appear to make modest interactions in these two enzyme systems, a different behavior from other enzymes investigated in this study. Therefore, the binding preference and stable interactions in these two systems may be related to non-catalytic residues. This behavior is very different than other enzyme systems such as human cyclophilin A, *E*. *coli* dihydrofolate reductase and horse liver alcohol dehydrogenase where the catalytic residues make the strongest interactions with the substrate [61–63].

**Table 2:** Averaged total interaction energy between nucleotide substrates and RNases. The total interaction energy (*E*_*total*_) was computed as a sum of van der Waals (*E*_*vdw*_) and electrostatics (*E*_*el*_) energy, averaged over 2,000 snapshots (see Methods for details). Difference >5 kcal/mol in *E*_*total*_ is considered significant for preference of substrate ACAC and AUAU binding to the same RNase; such cases are underlined. Note that the case of bRNaseA-ACAC at 300 K is close to this criterion (5.1 kcal/mol) but is not marked. The repulsive *E*_*vdw*_ observed in case of hRNase7-AUAU at 310 K is marked in bold.

	ACAC						AUAU					
	300 K			310 K			300 K			310 K		
	E_vdw_	E_el_	*E*_*total*_	E_vdw_	E_el_	*E*_*total*_	E_vdw_	E_el_	*E*_*total*_	E_vdw_	E_el_	*E*_*total*_
bRNaseA	-62.7	-34.4	*-97*.*1*	-59.1	-30.0	*-89*.*1*	-66.6	-35.5	*-102*.*2*	-61.4	-30.7	*-92*.*1*
hRNase1	-64.8	-36.2	*-101*.*0*	-69.0	-39.5	-*108*.*5*	-65.5	-35.9	*-101*.*4*	-64.1	-35.3	*-99*.*4*
hRNase2	-64.1	-44.0	*-108*.*1*	-59.4	-45.6	*-105*.*0*	-69.0	-36.3	*-105*.*2*	-69.9	-36.2	*-106*.*1*
hRNase3	-60.5	-51.9	*-112*.*3*	-51.9	-50.3	-*102*.*2*	-68.9	-43.1	*-111*.*9*	-56.7	-55.1	-*111*.*8*
hRNase4	-70.4	-45.6	*-116*.*0*	-66.9	-41.1	*-108*.*1*	-68.5	-34.4	*-102*.*9*	-69.9	-37.2	*-107*.*1*
hRNase5	-63.7	-36.6	*-100*.*3*	-55.9	-32.8	*-88*.*7*	-39.9	-8.4	*-48*.*3*	-51.9	-30.0	*-81*.*8*
hRNase6	-77.0	-39.8	*-116*.*8*	-79.4	-41.5	-*120*.*9*	-66.2	-38.1	-*104*.*3*	-74.8	-38.9	-*113*.*7*
hRNase7	-59.3	-41.9	*-101*.*2*	-66.7	-47.8	*-114*.*5*	-31.2	**11.3**	*-19*.*9*	-67.9	-42.7	*-110*.*5*

#### Dynamical characterization of the enzyme-substrate complexes

**Fig 7:**
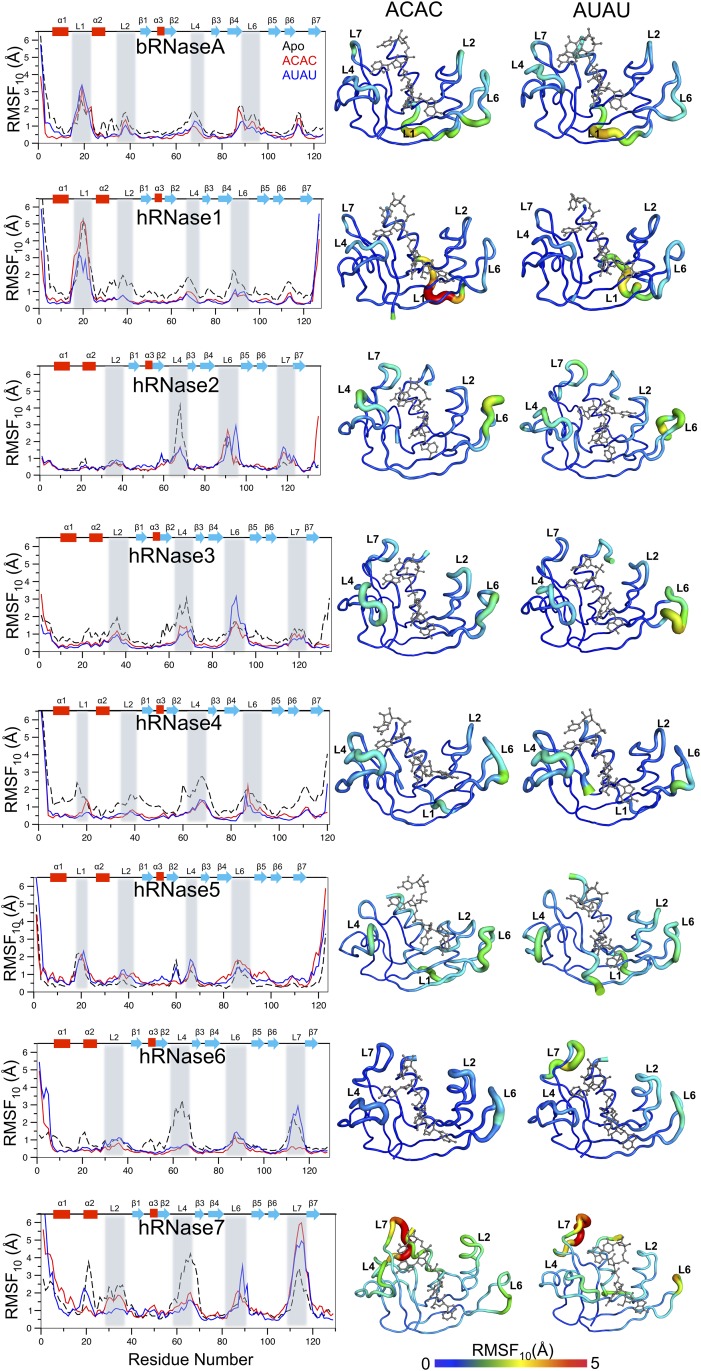
Dynamical behavior of RNases bound to model substrates at 300 K. In the left panel, for each enzyme-substrate [ACAC (red), AUAU (blue), and apo (black)], the flexibility of C_α_ atoms of each residue calculated from the slowest 10 quasi-harmonic modes (RMSF_10_) is shown as a function of enzyme residue numbering. Secondary structures [alpha-helix (α), beta sheets (β) and loops (L)] are identified (top); the important loop regions are highlighted in gray. On the right side, RMSF_10_ are projected on cartoon representations of each enzyme, with flexible loop regions identified. The thickness of the cartoon tube corresponds to the amplitude of RMSF_10_, and the color corresponds to the dynamic range observed: least dynamic (0 Å, blue) to most dynamic (5 Å, red). These fluctuations are on the same scale, allowing direct comparison between different enzymes and substrates. For clarity, fluctuations in the terminal regions are removed (bRNase 1–2; hRNase1 1–2 and 126–127; hRNase2 134–135; hRNase3 1–3; hRNase4 1–3; hRNase5 1–3 and 121–123; hRNase6 1–4; hRNase7 1–4) from the cartoon representation. Results are presented from simulations at 300 K. Results from 310 K simulations are shown in S15 Fig.

The conformational flexibility (or intrinsic dynamics) of the RNases in presence of the two model substrates was quantified using quasi-harmonic analysis (QHA) of the snapshots sampled during the MD simulations. The aggregated root means square fluctuation of top 10 slowest QHA modes (RMSF_10_) provides a good measure of a protein’s conformational flexibility. Such an analysis has been successfully used for relating dynamics to function for a number of other proteins [49, 64]. Fig 7 depicts the RMSF_10_ for the 8 enzymes when bound to the two model substrates, as well as enzymes without substrate (apo) at 300 K. The corresponding results from 310 K simulations are shown in S15 Fig. Results indicate that the most significant differences occur in the loop regions, similar to apo enzyme systems, as recently reported [30]. For RNases, and other enzymes, it is widely reported that motions of surface loops play important roles in function through various steps in the catalytic cycle [57, 58].

The dynamical characterization at 300 K indicates distinct differences in RNases flexibility upon ligand binding. The intrinsic dynamics of bRNaseA is very similar for the substrate-bound and the apo forms with the largest fluctuations observed for residues of loop L1 (residues 13–21). Note that bRNaseA has the highest catalytic activity among these 8 enzymes, and the long-range dynamics of loop L1 was previously shown to play a functional role in enzyme activity [5, 57]. hRNase1 showed overall reduction in fluctuations upon binding of the two substrates with additional reduction in fluctuations of loop L1 (residues 13–21) observed in the AUAU-bound state. The truncated loop L1 in hRNase2, hRNase3, hRNase6 and hRNase7 displayed diminished dynamics in these enzymes relative to bRNaseA. The large fluctuations in L4 (residues 58–71 for both enzymes) observed in the apo forms of hRNase2 and hRNase3 were diminished upon binding of the two substrates. Further, hRNase3 showed reduced conformational motions of L2 (residues 35–40) and L4 upon binding of the two substrates while loop 6 (residues 85–94) displayed enhanced motions upon binding of AUAU. hRNase4 showed significant reduction in the dynamical profile throughout the protein upon binding of the two substrates, consistent with similar changes in the conformational exchange patterns observed experimentally (data not shown). hRNase5 showed modest changes near L6 (residue 85–94) upon substrate binding. In the case of hRNase6, large fluctuations observed in L4 (residues 58–68) in the apo state were diminished upon binding of the two substrates. Further, the dynamics observed in loop L7 (residues 109–119) in the apo and AUAU-bound states was diminished in the ACAC-bound state. hRNase7 showed larger fluctuations upon binding of the two substrates in L7 (residues 109–119) while the reduced dynamics upon substrates binding was observed in L4 (residues 58–68).

In comparison to fluctuations observed at 300 K, the dynamical profiles at 310 K (S15 Fig) displayed minor differences in the fluctuations of loops of hRNase2, hRNase3 and hRNase4 for the two substrates. However, in the case of hRNase7, about a 2-fold increase in fluctuations are observed in loop L4 when bound to substrate ACAC, while the fluctuations in loop L7 are decreased for substrate AUAU at the higher temperature.

In a joint NMR-computational study of the RNase superfamily, we recently reported that the intrinsic dynamics of apo proteins is similar for members within phylogenetic sub-families that share common biological activities and different between sub-families [30]. Comparison of intrinsic dynamics when the two different substrates are bound also provides some unique insights. Comparison of members within phylogenetic subfamilies showed different dynamical properties upon binding of the two substrates. For example, AUAU binding led to reduced flexibility of L1 in hRNase1 relative to the apo state, while this change was not observed in bRNase1 at both 300 K and 310 K. Similarly, a comparison of hRNase2 and hRNase3, which belong to the same phylogenetic subfamily, suggests that while regions displaying large fluctuations are similar, there are notable differences in the changes in the dynamical profiles upon substrate binding between the two enzymes. These observations are consistent with recent studies which showed distinctly different effects of ligand binding for members within phylogenetic subfamilies [65]. However, further investigations are required, as the current work cannot fully rule out whether the observed changes in loop motions help in differentiating binding of substrates or are simply a result of substrate binding.

## Discussion

Ribonucleases are a superfamily of enzymes that catalyze the endonucleolytic cleavage of RNA substrates. The eight canonical human pancreatic-type RNases, identified in the initial sequencing of the human genome, perform distinct biological functions such as anti-pathogenicity, angiogenesis and host defense, among others [5, 6, 66]. In addition, they also share the common chemical function of ribonucleolytic activity of RNA substrates of non-specific nucleotide sequences. The human RNases investigated so far also display a diverse range of substrate specificities and catalytic efficiencies [5]. While numerous studies have characterized the nucleotide substrate binding properties, rate kinetics and efficiencies of human RNases, these studies were performed by different research groups with diverse substrates under various experimental conditions. Other studies also attempted to characterize the nucleotide preference based on available crystal structures [28]. Unfortunately, these comparisons also suffer the same limitations due to different substrates and varying conditions. These differences prevent a direct comparison of the nucleotide specificities across hRNases, which in turn limits our current understanding of the underlying mechanism for the observed nucleotide specificities as well as the detailed mechanism of biological and chemical function of these enzymes.

Characterizing the nucleotide binding properties of RNases with the same substrate(s) under identical conditions would provide a benchmark for comparison of members of this superfamily. This in turn would provide a framework for the systematic identification of structural and energetic contributions in the enzyme function. In this study, computer simulations and detailed analysis were used for characterization of human RNases 1–7 and bovine RNase A to probe the nucleotide binding properties in the presence of two model substrates–ACAC and AUAU. Differences in structural stability and hydrogen bonding interactions were characterized (Figs 3 and 4, Table 1, S1 and S2 Figs, and S1 Table). Further, the interaction energies between enzyme residues and substrate nucleotide pairs were calculated (as sum of van der Waals and electrostatics energy, see Fig 6 and Table 2). The results indicate that both AUAU and ACAC substrates stay either in or close to the binding pocket for most enzymes on the microsecond time-scale (Fig 2). hRNase 5 and hRNase7, however, show the weakest interactions with substrate AUAU. In one of the two alternate trajectories, the substrate was ejected from the binding pockets of these two enzymes, consistent with weaker binding affinities [67]. Note that we consider the set of two alternative trajectories as a representation of the expected behavior. A large set of alternate/longer trajectories (for each enzyme-substrate complex) will provide better quantitative estimates of how often the substrate is ejected out of the pocket; however, we expect the results presented here will be qualitatively similar to the results from the bigger set of trajectories.

Detailed description of model substrates ACAC and AUAU interactions with bRNaseA and hRNase1-7 are provided in supporting information text. Our results indicate that while all RNases used in this work share the conserved active-site catalytic triad (two histidine residues and one lysine), subtle changes in the binding site result in distinct binding interactions for the two substrates. Interestingly, the non-catalytic active-site residues show the strongest H-bonding interactions with the substrates. However, when the detailed interaction energies are calculated, some of these residues do not show the most favorable interactions; instead, the catalytic triad shows the most favorable interaction energy. One possible interpretation of these results is that rigid interactions are required between the enzymes and the substrates for stability. However, the interactions with the catalytic triad may need to be flexible for mechanistic aspects. In other words, strong H-bonds between the substrate nucleotides and non-catalytic active-site residues holds the substrate in place, while the catalytic residues with most favorable interactions need to be non-rigid. This is consistent with a number of previous reports, whereby dynamics of several conserved catalytic residues play an important role in the mechanism of bovine RNase A [5, 56, 57]. The results reported here require further comparison with experiments to confirm these observations.

Substrate preferences of bRNaseA and several human RNases were reported previously using a variety of RNA substrates [6, 9, 28]. A comparison of the H-bonding properties of the different RNases with the two model substrates AUAU and ACAC in this work suggest that bRNaseA and hRNase1 show similar H-bonding interactions with the two substrates. Interestingly, the H-bonding occupancy for hRNase2 and hRNase3 is significantly lower, consistent with the lower binding affinities and catalytic rates of these enzymes relative to bRNaseA at 300 K and 310 K. Interestingly, hRNase3 shows no interactions with the substrate ACAC at 310 K, suggesting a lower preference for this substrate. Similar results are observed for hRNase4 at 310K, which shows a significant increase in H-bonding occupancy for AUAU while a reduction in the occupancy is observed for ACAC-bound state. These observations for hRNase3 and hRNase4 at 310 K are consistent with the substrate preference reported for these enzymes [6, 9]. Our results further highlight the significant differences in the observed substrate binding properties of the different RNases between 300 K and 310 K. These observations suggest the importance of selecting temperatures for experimental and computational characterization of conformational properties and enzyme-substrate interactions that are representative of physiological conditions. As summarized in S1 Table, the majority of previous studies have been conducted at room temperature (298 K).

Overall, the results of this study show that all the 8 investigated RNase family members have conserved active-sites residues; however, there is not a uniform preference of a substrate across all of these enzymes. The difference in substrate preferences observed in this study are broadly consistent with previous observations using a variety of RNase substrates. While our results provide structural and energy-based insights that form the basis for this preference for the different pyrimidine bases, it is not clear how this relates to the diverse biological functions of these RNases. Further investigations are required to gain insights into sequence dependence on designated biological functions of host-defense (antibacterial and antiviral), angiogenesis and RNA cleavage.

## Supporting information

S1 File Supporting information for methods and results. This file contains additional text describing details of substrate complex model preparation, enzyme-substrate interactions calculations, a data set of PDB files corresponding to 64 MD trajectories, and description for the interaction energy analysis and summary of observations for the 8 systems.
(PDF)

S1 Table Summary of reported substrate binding affinities and enzyme activity among bovine and human pancreatic-type RNases. The binding affinity (*K*_M_), catalytic rate (*k*_cat_*)* and catalytic efficiency (*k*_cat_/*K*_M_) are presented, where available, for the different RNase members. Experiments have used substrate with phosphate groups before or after terminal nucleotides. Here, A, C, G and U indicate nucleosides and phosphate groups are explicitly indicated as p. Poly refers to RNA sequences with repeats of same nucleotide. > denotes cyclic nucleotide substrates. tRNA denotes substrate of non-specific sequence obtained from cellular tRNA, ytRNA indicates tRNA from yeast. The source of data is indicated in the last column. Temperature used for the kinetics studies is provided in Kelvins.
(DOCX)

S2 Table H-bonding properties of bovine and human pancreatic-type RNases at 310 K. The H-bonds that meet the criteria of 3.3 Å, angle between 135–180° and more than 45% occupancy are listed. Note that the results are based on analysis of both MD alternate trajectories for each system (see Methods for details).
(DOCX)

S3 Table Residues showing significant favorable interactions with the substrates, at 300 K. An interaction is considered as significant if the enzyme-nucleotide pair with interaction energy < -3 kcal/mol of *E*_*total*_. Results for central two nucleotides and terminal nucleotides are shown separately. The enzyme residues are listed in the order of decreasing energy of interaction with the nucleotide making the favorable interaction is listed after value.
(DOCX)

S4 Table Important residues showing significant favorable interactions with the substrates, at 310 K. An interaction is considered as significant if the enzyme-nucleotide pair with interaction energy < -3 kcal/mol of *E*_*total*_. Results for central two nucleotides and terminal nucleotides are shown separately. The enzyme residues are listed in the order of decreasing energy of interaction with the nucleotide making the favorable interaction is listed after value.
(DOCX)

S1 Fig Sequence alignment of hRNases 1–7 and bovine RNase A. (TIF)

S2 Fig The equilibration protocol used allows careful adjustment of overlapping protein residues and nucleotide substrate. A. Starting conformation, where residue Gln117 has steric clashes with the modeled substrate. B. Ending conformation after the equilibration protocol, where the steric clash between the Gln117 and substrate has been fully resolved.
(TIF)

S3 Fig Substrate behavior over the course of 0.5 μs MD simulations at 310 K. The enzymes are shown as gray cartoons and the 3 relative positions of the substrate at start (0 μs, green), mid-way (0.25 μs, yellow) and end (0.5 μs, magenta) of the simulations are shown as sticks. The results for the two substrates ACAC and AUAU are shown separately. The central two nucleotides of the substrate that interact with two binding sub-sites (B_1_/P_1_ or B_2_/P_2_) are shown in dark colors, while the two terminal nucleotides (one on each side) are depicted with faded color. The results depicted are from the 310 K simulation trajectories and represent the MD simulations set 1. The results from trajectory 2 at 310 K are qualitatively similar (Table 2).
(TIF)

S4 Fig Hydrogen-bonding interactions between RNases and model substrates ACAC and AUAU at 310 K. H-bonds (with >45% occupancy) are indicated with black dotted line and averaged bond length (Å) and percentage occupancy are shown in red colored text. Enzyme and nucleotide atoms participating in the H-bonds are labeled, with enzyme residues shown in blue and substrate nucleotides shown in green for carbon atoms, blue for nitrogen, red for oxygen, and orange for phosphorus. Enzyme residue numbers correspond to enzyme sequence, while substrate nucleotides are numbered as A_(-2)_C/U_(-1)_A_(1)_C/U_(2)_, as described in the legend of Fig 1. Note that the substrates orientation (marked by 3’ and 5’ for bRNaseA) appear opposite to the depiction in Fig 1, as enzymes are shown from view used commonly in literature. The percentage occupancies are rounded off to the nearest whole number.
(TIF)

S5 Fig Important H-bond behavior over time for bRNaseA-substrate complexes. Results from ACAC are shown on top, and from AUAC complex on the bottom. The two alternate trajectories are marked as (1) and (2) for two temperatures 300 K and 310 K. Red lines mark the distance range used for defining H-bonds.
(TIF)

S6 Fig Important H-bond behavior over time for hRNase1-substrate complexes. Results from ACAC are shown on top, and from AUAC complex on the bottom. The two alternate trajectories are marked as (1) and (2) for two temperatures 300 K and 310 K. Red lines mark the distance range used for defining H-bonds.
(TIF)

S7 Fig Important H-bond behavior over time for hRNase2-substrate complexes. Results from ACAC are shown on top, and from AUAC complex on the bottom. The two alternate trajectories are marked as (1) and (2) for two temperatures 300 K and 310 K. Red lines mark the distance range used for defining H-bonds.
(TIF)

S8 Fig Important H-bond behavior over time for hRNase3-substrate complexes. Results from ACAC are shown on top, and from AUAC complex on the bottom. The two alternate trajectories are marked as (1) and (2) for two temperatures 300 K and 310 K. Red lines mark the distance range used for defining H-bonds.
(TIF)

S9 Fig Important H-bond behavior over time for hRNase4-substrate complexes. Results from ACAC are shown on top, and from AUAC complex on the bottom. The two alternate trajectories are marked as (1) and (2) for two temperatures 300 K and 310 K. Red lines mark the distance range used for defining H-bonds.
(TIF)

S10 Fig Important H-bond behavior over time for hRNase5-substrate complexes. Results from ACAC are shown on top, and from AUAC complex on the bottom. The two alternate trajectories are marked as (1) and (2) for two temperatures 300 K and 310 K. Red lines mark the distance range used for defining H-bonds.
(TIF)

S11 Fig Important H-bond behavior over time for hRNase6-substrate complexes. Results from ACAC are shown on top, and from AUAC complex on the bottom. The two alternate trajectories are marked as (1) and (2) for two temperatures 300 K and 310 K. Red lines mark the distance range used for defining H-bonds.
(TIF)

S12 Fig Important H-bond behavior over time for hRNase7-substrate complexes. Results from ACAC are shown on top, and from AUAC complex on the bottom. The two alternate trajectories are marked as (1) and (2) for two temperatures 300 K and 310 K. Red lines mark the distance range used for defining H-bonds.
(TIF)

S13 Fig Electrostatic surface of RNases with the two model substrates at 310 K. Enzyme conformation from the MD ensemble closest to the averaged structure for the entire trajectory (conformation with smallest RMSD with averaged structure) was used to calculate the representative electrostatic potential (+5kT/e in blue and –5kT/e per electron in red).
(TIF)

S14 Fig Enzyme-substrate interaction energy at 310 K. Total interaction energy was computed as a summation of electrostatic and van der Waals interaction energy between atom pairs of enzyme residue and substrate nucleotide (ACAC and AUAU, respectively). The areas of largest negative energy (blue) represent favorable interactions, while positive energy (red) represents less favorable or unfavorable interactions. For ease of comparison, sequence of the model substrate has been adjusted to match the orientation depicted in Figs 2 and 3 (CACA and UAUA). Enzyme residues showing total interaction energy (*E*_*total*_, S3 Table) contributions < -3 kcal/mol were considered significant and are marked with ellipses.
(TIF)

S15 Fig Dynamical behavior of RNases bound to model substrates at 310 K. In the left panel, for each enzyme-substrate [ACAC (red), and AUAU (blue), the flexibility of C_α_ atoms of each residue calculated from the slowest 10 quasi-harmonic modes (RMSF_10_) is shown as a function of enzyme residue numbering. Secondary structures [alpha-helix (α), beta sheets (β) and loops (L)] are identified (top); the important loop regions are highlighted in gray. On the right side, RMSF_10_ are projected on cartoon representations of each enzyme, with flexible loop regions identified. The thickness of the cartoon tube corresponds to the amplitude of RMSF_10_, and the color corresponds to the dynamic range observed: least dynamic (0 Å, blue) to most dynamic (5 Å, red). These fluctuations are on the same scale, allowing direct comparison between different enzymes and substrates. For clarity, fluctuations in the terminal regions are removed (bRNase 1–2; hRNase1 1–2 and 125–127; hRNase4 1–3 and 119–120; hRNase5 1–2 and 121–123; hRNase6 1–3; hRNase7 1–3) from the cartoon representation.
(TIF)
