# Supplementary material for: Nucleotide substrate binding characterization in human pancreatic-type ribonucleases
Source: PLoS One. 2019 Aug 8;14(8):e0220037. doi: 10.1371/journal.pone.0220037 (PMC6687278; doi:10.1371/journal.pone.0220037)
Supplement: S1 File — This file contains additional text describing details of substrate complex model preparation, enzyme-substrate interactions calculations, a data set of PDB files corresponding to 64 MD trajectories, and description for the interaction energy analysis and summary of observations for the 8 systems. (PDF) [file pone.0220037.s001.pdf]

METHODS

Detailed methodology for enzyme-substrate complexes: For preparation of the enzyme-substrate complexes, the enzyme structure was aligned to the protein residues of bRNaseA available in the 1RCN PDB structure (using PyMOL's align feature). Based on the relative orientation, the phosphate backbone and the ribose sugar coordinates for DNA ligand (ATAA), from the PDB structure 1RCN, were used for the RNA substrate modeling. In addition, the coordinates of full nucleotide bases were also used for the two A nucleotides, while the nucleotide base coordinates for C and U were added by AMBER's *leap* module based on the templates available within the AMBER library by aligning the phosphate backbone and ribose sugar coordinates.

The equilibration protocol used has carefully designed to resolve any overlaps or clashes that occur between the protein and positioned substrate. For example, hRNase 5 has Gln117 which occupies the active-site. The super-positioning of the substrate based on alignment of protein structure of hRNase5 with bRNaseA leads to overlap between Gln117 and the model substrate. The equilibration protocol fully resolves this overlap and steric clashes (Figure S2). At the start of the equilibration Gln117 is overlapping with coordinates of substrate nucleotide (Figure S2A) and after the 16-step careful equilibration protocol (fully described in *Proteins: Structure, Function, and Bioinformatics* **2004**, 56 (3), 449-463) the side chain of Gln117 adopted another conformation to accommodate the substrate (Figure S2B). Note that at the end of

equilibration process, there were no restraints or constraints in place, still the conflict and steric clash between Gln117 and the nucleotide substrate has been fully resolved.

Detailed methodology for computing enzyme-substrate interactions: The energy for the enzyme-substrate interactions ($E_{enz\text{-}subs}$) were calculated as a sum of electrostatic and van der Waals energy between atom pairs.

$$E_{enz-subs} = \sum (E_{el} + E_{vdw}) \quad (1)$$

E_{el} is the electrostatic contribution, E_{vdw} is the van der Waals term and the summation runs over all atom pairs for the enzyme and nucleotide substrate. The E_{el} and E_{vdw} terms were computed as follows

$$E_{el} = \frac{q_i q_j}{\varepsilon(r) r_{ij}} \quad \text{and} \quad E_{vdw} = \frac{A_{ij}}{r_{ij}^{12}} - \frac{B_{ij}}{r_{ij}^{6}} \quad (2)$$

where q_i , q_j are partial charges, and A_{ij}, B_{ij} are Lennard-Jones parameters. These parameters were obtained from AMBER *ff14SB* force field. A distance-dependent dielectric function was used:

$$\varepsilon(r_{ij}) = A + \frac{B}{1 + k \exp(-\lambda B r_{ij})} \quad (3)$$

B=ε_o-A; ε_o=78.4 for water; A= -8.5525; λ=0.003627 and k=7.7839.

RESULTS

MD trajectories from set 1 and 2

The 64 MD trajectories for the simulation set 1 and 2 (summarized in Table 1) are available in PDB at: https://doi.org/10.17026/dans-237-qxmh

This data set contains all the 8 systems including human ribonucleases 1-7 and bovine ribonuclease A, in complex with two model nucleotide substrates ACAC and AUAU. The simulations where performed at two temperatures 300 K and 310 K, and in duplicates (two trajectories). The duration of the trajectories is 500 nanoseconds (ns). Larger files have been split in two parts.

Details of interaction energy analysis

bRNaseA: More specifically, in the case of bRNaseA Lys7, Val43, Thr45, His119 and Phe120 show the most favorable interaction energies with both ACAC and AUAU substrates. Asn44 and Lys66 are other residues that further contribute to binding of substrate ACAC, while Lys41 contributes to binding with AUAU. At the higher temperature, 310 K, substrate ACAC interacts with the same residues of bRNaseA as at 300 K. However, quantitative comparison indicates that Lys66 and Asn71 have favorable interaction energy with AUAU, while the interaction energy for Lys7 and Lys41 is reduced when AUAU binds to bRNaseA at higher temperature.

hRNase1: In the case of hRNase1, Arg4, Val43, Thr45, Lys66, His119, and Phe120 make large contributions to interaction energies with both substrates, similar to bRNaseA; furthermore, substrate AUAU is observed to have favorable interaction energies with

additional residues (Lys7, Asn44 and Asn71). Similar interactions are observed for hRNase1 with both substrates at the higher temperature.

hRNase2: Residues of hRNase2 that show favorable interaction energies with both the substrates are Lys39, Gln41, Thr43 and His130. Residues Asn42 and Arg69 contribute additionally to the binding of substrate ACAC, while residues Trp8, Leu131 and Arg133 contribute to binding with substrate AUAU. Further, at higher temperature the interactions of substrate ACAC with residues Asn42, Thr43, and Arg69 are reduced while the interactions of residues Arg37 and Thr43 are reduced with substrate AUAU, which is a different behavior relative to bRNaseA and hRNase1. However, residues Met1 and Arg133 of hRNase2 have favorable interaction energies when binding to substrate ACAC and AUAU respectively, at higher temperature 310 K.

hRNase3: In the case of hRNase3, Lys39 and His129 make the largest contributions towards interaction with the two substrates. Leu130 is observed to have additional contributions to interaction energy in the case of substrate AUAU. At higher temperature, the N-terminal residue Met1 of hRNase3 makes favorable contributions to interaction energies with both substrates. Substrate ACAC is also observed to make additional favorable interactions with Arg2, Arg8 and Arg35, while the interactions of residue Leu130 with substrate AUAU is reduced at higher temperature.

hRNase4: In the case of hRNase4, His117 and Phe118 are the largest contributors to interaction energies with both substrates. Residues Arg8, Lys41 and Thr45 also make

strong favorable interactions with both the substrates. Phe43 and Lys66 have additional favorable interactions with AUAU and ACAC respectively. Furthermore, at higher temperature 310 K, similar interaction profile was observed for both substrates.

hRNase5: hRNase5 residues Lys40 and His114 have favorable interactions with both substrates, while residues Thr44, Leu115, Gln117 and Arg121 have favorable interaction energy only with substrate ACAC. At 310 K, in contrast to other systems, the interactions of both substrates with hRNase5 changes considerably. Residues Ile42, Asn43 and Phe120 make additional strong interactions with substrate ACAC, while strong interaction energies with residues Gln117 and Arg121 are reduced. The number of residues having favorable interactions energies with substrate AUAU at higher temperature increases significantly when compared to 300 K, which includes Arg5, His13, Asn43, Leu115 and Gln117. Note that Gln117 was previously suggested as the residue that partially blocks active-site entrance for optimal substrate binding to hRNase5, thus significantly affecting catalysis in this family member (34).

hRNase6: hRNase6 residues Asn65, Arg67 Asn69 and His123, Leu124, make the most significant contributions to the interaction energies with both substrates. Strong interactions of ACAC are observed with Lys8, Trp11, Gln15, His16 and Lys39. On the other hand, Lys64 is observed to strongly interact with AUAU only at 300 K. At higher temperature, strong interactions of these residues with both substrates are preserved with additional contributions from residues Gln15, Thr43, Asn65, Arg67 and Asn69 for substrate ACAC and Lys8 and Thr43 for substrate AUAU.

hRNase7: In the case of hRNase7, His124 is the largest contributor to the interaction energy for both substrates. Lys39, Leu41, Asn42 and Thr43 are observed to make additional contributions to interaction energies only with substrate ACAC. At the higher temperature, strong interactions of hRNase7 residues with substrate ACAC are preserved with additional contribution from residue Leu125. However, at 310 K, the number of residues making strong interactions with the substrate AUAU increases, these include Met1, Lys39, Lys64 and Leu125.

Summary of observations for each RNases investigated in this study

bRNaseA: Both the substrates ACAC and AUAU show stable binding in the active-site on the microsecond time-scale. The substrates show strong H-bond interactions with residues Thr45, Asp71, and Phe120. In addition, Gln11 shows H-bond interaction with substrate AUAU. The dynamical motions of the enzyme are similar in the case of two substrates, exhibiting highest flexibility in loops L1 and L6. The electrostatic surface of the bRNaseA is mostly positively charged but shows slight differences in the case of ACAC and AUAU, which appear to be an anomaly for the set of proteins investigated in this study. The residue Lys7, Val43, Thr45, Lys66, His119, and Phe120 show large favorable van der Waals and electrostatic interactions with these substrates. While the dynamical behavior at the temperatures 300 K and 310 K are qualitatively similar for both substrates, the overall strength of the favorable enzyme-substrate interactions increase at higher temperature (corresponding to more negative energy in Table 4); however, the number of H-bond interactions with substrate AUAU are reduced at 310 K.

hRNase1: Similar to the case on bRNaseA, the two substrates ACAC and AUAU show stable binding in the active-site of hRNase1 on the microsecond time-scale. However, the substrate ACAC forms strong H-bond interactions only with Thr45, while AUAU is observed to form strong H-bonds with Thr45, Asn71 and Phe120. The electrostatic surface of hRNase1 is mostly positively charged and very similar in the case of both substrates. Residues Arg4, Val43, Thr45, Lys66, His119 and Phe120 show the most favorable van der Waals and electrostatic interactions with both substrates. The dynamical motions of the enzyme are very similar in the case of two substrates, with large flexibility in loop L1; however, this loop shows higher flexibility when bound to ACAC than AUAU. An increase in number of H-bonds and their occupancies is observed at higher temperature (310 K) for both substrates investigated. The electrostatic and van der Waals interactions are more favorable for substrate ACAC and less favorable for substrate AUAU at the higher temperature investigated. The dynamical behavior at the temperatures 300 K and 310 K are similar in presence of both substrates.

hRNase2: Substrate AUAU shows stable binding in the hRNase2 active site at both temperatures, while the substrate ACAC shows stable binding only at 300 K. However, strong hydrogen bonds are observed with residues Gln15 and Thr43 in both cases. hRNase2 has mostly positively electrostatic surface when bound to both substrates as well. The van der Waals and electrostatic interactions show that residues Lys39, Gln41, Thr43 and His130 make the largest favorable contributions to interaction energy with both substrates. The dynamical motions of the enzyme are observed to be very similar for the two substrates, with high flexibility in loops L4, L6 and L7. The dynamical behavior

is mostly similar at the two temperatures, except for loop L7 where increased fluctuations are observed with substrate AUAU at 310 K. The H-bond occupancies with substrate ACAC is reduced at 310 K as compared to simulations at 300 K. The interactions for substrate ACAC are weakened at higher temperature while it remains the same for AUAU at both temperatures.

hRNase3: Both the substrates ACAC and AUAU show significantly less stability in the active-site of hRNase3 on the microsecond time-scale as compared to the other members of the family investigated in this study. Only residue Asn71 (and Thr133 for AUAU) shows hydrogen bonding interactions with the substrates. Much like other RNases, the electrostatic surface of the hRNase3 shows the presence of mostly positively charged residues and is also similar in the case of both substrates investigated. Residues Lys39 and His129 make the largest contribution to van der Waals and electrostatic interaction energy. The protein dynamics is very similar for the two substrates, with largest flexibility in loops L2, L4 and L6; however, loop L6 has higher fluctuation when bound to AUAU than ACAC. At 310 K, no strong H-bonds are observed with substrate ACAC and the interactions become less favorable; however, the H-bond occupancies for substrate AUAU are considerably reduced, and the overall interaction energy remains the same. Dynamics of the enzyme remains the same for most regions at the two temperatures for both substrates.

hRNase4: On the microsecond time-scale, both substrates are mostly stable in the active-site of hRNase4. Substrate ACAC forms strong hydrogen bonds with residues Thr45,

Asn71, His117 and Phe118; while substrate AUAU forms hydrogen bonds only with Thr45 and Phe118. The electrostatic surface of hRNase4 is also mostly positively charged and similar in the case of both substrates. His117 and Phe118 are the largest contributors to van der Waals and electrostatic interaction energy with the two substrates. Lys8, Lys41 and Thr45 also make large contributions towards electrostatic and van der Waals interaction energy for both substrates. The dynamical motions are observed to be very similar for the two substrates. While the dynamical behavior is similar at the two temperatures, considerable difference is observed in H-bond occupancies. At higher temperature, the number of H-bonds with substrate ACAC is reduced significantly, whereas an increase is observed in the H-bonds and their occupancies with substrate AUAU, consistent with decrease in the interaction energy (less negative value in Table 4) in case of ACAC but increase in the case of AUAU. Further, these observations are consistent with the preference of the uridine nucleotide in the B_1 sub-site reported previously for hRNase4 (10).

hRNase5: For hRNase5, substrate ACAC is more stable in the active-site than the substrate AUAU on the microsecond time-scale. In one of the trajectories investigated, the substrate AUAU is completely ejected out of the active-site. This behavior is different than other hRNases (except for hRNase7, as discussed below), where either both or one central nucleotide of the substrate stays bound in the active-site. This is also indicated by the observation that substrate ACAC forms strong hydrogen bonds with Thr44 and His114, while no hydrogen bonds are observed between substrate AUAU and the enzyme. Much like other RNases in this study, the electrostatic surface of hRNase5 is

also mostly positively charged. Residues Lys40 and His140 make the largest contribution to van der Waals and electrostatic interaction energy. The dynamical motions are observed to be very similar for the two substrates with flexibility in loops L1, L2 and L6 when bound to both substrates. The dynamical behavior at two temperatures 300 K and 310 K is similar. At the higher temperature, H-bonds are formed with substrate AUAU, contrary to the observations at 300 K. Additionally, an increase in the favorable interaction is observed, while in the case of substrate ACAC, diminished interactions are observed.

hRNase6: Both the substrates ACAC and AUAU are stable in the hRNase6 active-site on the microsecond time-scale. Residue Gln15, Asn69 and Trp11 form strong hydrogen bonds with substrate ACAC, while substrate AUAU shows strong hydrogen bonding with Asn65, Arg67 and Asn69. The electrostatic surface of hRNase6 is mostly positively charged and similar in the case of both substrates. The van der Waals and electrostatic interactions show that His123 and Leu124 make the largest favorable contributions to interaction energy in case of both substrates. Additionally, large contributions are also observed form residues Asn65, Arg67 and Asn69 in presence of both substrates. The dynamical motions are very similar for the two substrates, except for loop L7 that shows higher fluctuations when bound to AUAU than ACAC. The dynamical behavior and hydrogen bond occupancies are alike at the two temperatures, while the favorable interactions become more pronounced at higher temperature for both substrates.

hRNase7: The substrates ACAC and AUAU are less stable in interacting with the active-site of hRNase7, forming only one strong hydrogen bond with Thr43 and Leu125, respectively. Similar to the case of hRNase7, in one of the trajectories investigated, substrate AUAU is completely ejected out of the active-site. The electrostatic surface of hRNase7 appears more positively charged when substrate ACAC is bound when compared to AUAU. Residues Lys39 and His124 make the largest contribution to van der Waals and electrostatic interaction energy. The enzyme dynamics is observed to be different for the two substrates with hRNase7: loop L2 and L7 show higher fluctuations when bound to ACAC; while loop L6 shows higher fluctuations when bound to AUAU. The overall dynamics of hRNase7 at two temperatures 300 K and 310 K is observed to be similar, except for loop L4 that shows higher fluctuation when substrate ACAC is bound. However, considerable change is observed in H-bond patterns at the two temperatures investigated. At higher temperature, more H-bonds with higher occupancies are formed with substrate ACAC, while no strong H-bonds are formed with substrate AUAU. Also, considerable increase in the favorable interaction energy is observed for both substrates at 310 K.
