# Supplementary material for: Nucleotide substrate binding characterization in human pancreatic-type ribonucleases
Source: PLoS One. 2019 Aug 8;14(8):e0220037. doi: 10.1371/journal.pone.0220037 (PMC6687278; doi:10.1371/journal.pone.0220037)
Supplement: S1 Table — The binding affinity (KM), catalytic rate (kcat) and catalytic efficiency (kcat/KM) are presented, where available, for the different RNase members. Experiments have used substrate with phosphate groups before or after terminal nucleotides. Here, A, C, G and U indicate nucleosides and phosphate groups are explicitly indicated as p. Poly refers to RNA sequences with repeats of same nucleotide. > denotes cyclic nucleotide substrates. tRNA denotes substrate of non-specific sequence obtained from cellular tRNA, ytRNA indicates tRNA from yeast. The source of data is indicated in the last column. Temperature used for the kinetics studies is provided in Kelvins. (DOCX) [file pone.0220037.s002.docx]

**Table ST1**

| **Protein** | **Substrate** | | ***K_M_* (mM)** | ***k_cat_* (s^-1^)** | ***k_cat_*/*K_M_* (s^-1^M^-1^) x 10^6^** | **Temp. (K)** | **Ref.** |
| --- | --- | --- | --- | --- | --- | --- | --- |
| bRNaseA | UpA | | 0.20 ± 0.06 | 347 ± 40 | 1.7 ± 0.3 | 298 | ^10^ |
| bRNaseA | UpA | | 0.33 | 1920 | 5.8 | 298 | ^11^ |
| bRNaseA | UpA | | 0.62 ± 0.09 | 1400 ± 150 | 2.3 ± 0.4 | 298 | ^12^ |
| bRNaseA | UpA | | 0.7 | 2690 | 3.0 ± 0.1 | 298 | ^13^ |
| bRNaseA | CpA | | 0.5 | 2300 | 6.4 ± 0.7 | 298 | ^13^ |
| bRNaseA | C > p | | 1.06±0.1 | 2.28±0.18 | 0.00215 | 298 | ^9^ |
| bRNaseA | Poly(C) | | 0.047 ± 0.011 | 190 ± 11 | 4.0 ± 1.0 | 283 | ^14^ |
| bRNaseA | Poly(C) | | 0.089 ± 0.009 | 507 ± 15 | 5.7 ± 0.5 | 298 | ^10^ |
| bRNaseA | Poly(C) | | 0.0331 ± 2.6 | 368.0 ± 11.0 | 11.2 ± 0.6 |  | ^15^ |
| bRNaseA | Poly(U) | | 0.06 | 24 | 0.4 | 298 | ^11^ |
| bRNaseA | Poly(C) | | 0.034 | 510 | 15 | 298 | ^11^ |
| bRNaseA | Poly(U) | | 0.06 ± 0.01 | 24 ± 15 | 0.4 ± 0.3 | 298 | ^12^ |
| bRNaseA | Poly(C) | | 0.034 ± 0.002 | 510 ± 10 | 15 ± 1 | 298 | ^12^ |
| bRNaseA | Poly(A) | | 0.080 ± 0.009 | 0.023 ± 0.001 | 0.00028 ± 0.00004 | 298 | ^12^ |
|  | |  | | | | | |
| hRNase1 | Poly(C) | | 0.10 ± 0.013 | 2416.67 ± 33.3 | 1.48 ± 0.11 | 310 | ^16^ |
| hRNase1 | tRNA | | 4.0 | 64.8 | 0.0.162 |  | ^17^ |
|  | |  | | | | | |
| hRNase2 | tRNA | | 0.0007 | 0.91 | 0.13 |  | ^18^ |
| hRNase2 | tRNA | | 0.00336 ± 0.00015 | 31.37 ± 0.96 | 9.34 ± 0.17 |  | ^19^ |
| hRNase2 | tRNA | | 0.0128 | 3.7 | 3.2 | 310 | ^20^ |
| hRNase2 | Poly(U) | | 80 ± 7 | 190 ± 11 |  |  | ^19^ |
| hRNase2 | tRNA | | 2.5 ± 0.21 | 20 ± 0.65 |  |  | ^19^ |
| hRNase2 | tRNA | | 0.0036 ± 0.00015 | 31.37 ± 0.96 | 9.34 ± 0.17 |  | ^21^ |
| hRNase2 | Poly(U) | | 53.05 ± 4.04 | 1248.93 ± 12.10 | 4.79 ± 0.52 |  | ^21^ |
|  | |  | | | | | |
| hRNase3 | CpA | | 1.7±0.3 | 0.55±0.06 | 0.000323 | 298 | ^9^ |
| hRNase3 | UpA | | 2.7±0.66 | 1.22±0.12 | 0.000447 | 298 | ^9^ |
| hRNase3 | C > p | | 3±0.53 | 0.0032±0.00051 | 1.07×10^−6^ | 298 | ^9^ |
| hRNase3 | C > p | | 1.5 | 0.014 | 0.000011 | 298 | ^22^ |
| hRNase3 | U > p | | 1.0 | 0.0043 | 0.000004 | 298 | ^22^ |
| hRNase3 | CpA | | 2.4 | 4.2 | 0.001750 | 298 | ^22^ |
| hRNase3 | UpA | | 5.4 | 6.2 | 0.001150 | 298 | ^22^ |
| hRNase3 | (Up)2 U > p | | 1.4 | 0.56 | 0.000400 | 298 | ^22^ |
| hRNase3 | (Up)3 U > p | | 0.7 | 1.2 | 0.001714 | 298 | ^22^ |
| hRNase3 | (Up)4 U > p | | 0.17 | 1.4 | 0.008235 | 298 | ^22^ |
| hRNase3 | ytRNA | | 0.0041 | 0.0024 | 0.000590 | 298 | ^22^ |
| hRNase3 | Poly(U) | | 253 ± 10 | 36 ± 0.4 | 0.00014 ± 0.000005 |  | ^23^ |
|  | |  | | | | | |
| hRNase4 | UpA | |  |  | 0.25 | 298 | ^24^ |
| hRNase4 | CpA | |  |  | 0.00066 |  |  |
|  | |  | | | | | |
| hRNase5 | CpA | |  |  | 0.0000122 ± 0.0000002 | 298 | ^25,26^ |
| hRNase5 | CpG | |  |  | 0.000004 ± 0.0000001 | 298 | ^25,26^ |
| hRNase5 | CpC | |  |  | 0.0000013 ± 0.0000001 | 298 | ^25,26^ |
| hRNase5 | CpU | |  |  | 0.0000006 ± 0.0000001 | 298 | ^25,26^ |
| hRNase5 | UpA | |  |  | 0.0000007 ± 0.0000001 | 298 | ^25,26^ |
| hRNase5 | UpG | |  |  | 0.00000013 ± 0.00000002 | 298 | ^25,26^ |
| hRNase5 | UpC | |  |  | 0.00000006 ± 0.00000001 | 298 | ^25,26^ |
| hRNase5 | UpU | |  |  | 0.000000031 ± 0.000000007 | 298 | ^25,26^ |
| hRNase5 | pCpA | |  |  | 0.000019 ± 0.000001 | 298 | ^25,26^ |
| hRNase5 | CpAp | |  |  | 0.000110 ± 0.000005 | 298 | ^25,26^ |
| hRNase5 | CpApG | |  |  | 0.000055 ± 0.000002 | 298 | ^25,26^ |
| hRNase5 | CpApA | |  |  | 0.000044 ± 0.000002 | 298 | ^25,26^ |
| hRNase5 | CpApU | |  |  | 0.000030 ± 0.000001 | 298 | ^25,26^ |
| hRNase5 | CpApC | |  |  | 0.000021 ± 0.000001 | 298 | ^25,26^ |
| hRNase5 | CpA | |  |  | 0.0000024 ± 0.0000001 | 298 | ^25,26^ |
| hRNase5 | CpAp | |  |  | 0.000017 ± 0.0000001 | 298 | ^25,26^ |
| hRNase5 | CpApG | |  |  | 0.0000117 ± 0.0000003 | 298 | ^25,26^ |
| hRNase5 | CpA | |  |  | 0.0000040 ± 0.0000001 | 298 | ^25,26^ |
| hRNase5 | CpAp | |  |  | 0.000033 ± 0.0000002 | 298 | ^25,26^ |
| hRNase5 | CpApG | |  |  | 0.000016 ± 0.0000004 | 298 | ^25,26^ |
|  | |  | | | | | |
| hRNase6 | tRNA | | 0.005 | 0.13 | 0.000026 | 298 | ^9,31^ |
| hRNase6 | UpA | | 2.63±0.3 | 12.9±1.1 | 0.0049 | 298 | ^9^ |
| hRNase6 | CpA | | 1.22±0.2 | 1.08±0.1 | 0.000885 | 298 | ^9^ |
| hRNase6 | C>p | | 2.06±0.3 | 0.00325 ± 0.06 | 1.6 ×10^−6^ | 298 | ^9^ |
|  |  | |  |  |  |  |  |
| hRNase7 | ytRNA | | 0.0022 | 5.1 | 2.3 | 310 | ^27^ |
|  |  | |  |  |  |  |  |
