# Supplementary material for: Nucleotide substrate binding characterization in human pancreatic-type ribonucleases
Source: PLoS One. 2019 Aug 8;14(8):e0220037. doi: 10.1371/journal.pone.0220037 (PMC6687278; doi:10.1371/journal.pone.0220037)
Supplement: S2 Table — The H-bonds that meet the criteria of 3.3 Å, angle between 135–180° and more than 45% occupancy are listed. Note that the results are based on analysis of both MD alternate trajectories for each system (see Methods for details). (DOCX) [file pone.0220037.s003.docx]

**Table ST2**

|  | **ACAC** | | | **AUAU** | | |
| --- | --- | --- | --- | --- | --- | --- |
|  | **Substrate** | **Residue** | **bond length**  **(% occupancy)** | **Substrate** | **Residue** | **bond length**  **(% occupancy)** |
| **bRNaseA** | C_(-1)_ (N_3_)  C_(-1)_ (O_2_)  A_(1)_ (N_1_)  C_(-1)_ (O_2'_)  A_(1)_ (N_6_) | Thr45 (O_ϒ1_)  Thr45 (N)  Asn71 (N_δ2_)  Phe120 (O)  Asn71 (O_δ1_) | 2.88 (91.4%)  2.88 (86.4%)  3.02 (58.7%)  2.74 (48.7%)  2.99 (46.3%) | A_(1)_ (N_1_)  U_(-1)_ (O_2_)  A_(1)_ (N_6_)  U_(-1)_ (N_3_) | Asn71 (N_δ2_)  Thr45 (N)  Asn71 (O_δ1_)  Thr45 (O_ϒ1_) | 3.02 (79.2%)  2.91 (78.8%)  2.96 (76.8%)  2.93 (74.9%) |
| **hRNase1** | C_(-1)_ (N_3_)  C_(-1)_ (O_2_)  A_(1)_ (N_1_)  A_(1)_ (N_6_)  C_(-1)_(O_2'_) | Thr45 (O_ϒ1_)  Thr45 (N)  Asn71 (N_δ2_)  Asn71 (O_δ1_)  Phe120 (O) | 2.87 (93.6%)  2.88 (84.4%)  3.02 (70.1%)  2.98 (61.2%)  2.74 (45.4%) | U_(-1)_ (N_3_)  A_(1)_ (N_1_)  U_(-1)_ (O_2_)  A_(1)_ (N_6_)  U_(-1)_ (O_2'_) | Thr45 (O_ϒ1_)  Asn71 (N_δ2_)  Thr45 (N)  Asn71 (O_δ1_)  Phe120 (O) | 2.92 (93.9%)  3.00 (80.0%)  2.91 (76.9%)  2.97 (73.3%)  2.73 (52.0%) |
| **hRNase2** | A_(1)_ (O_P2_)  C_(-1)_ (N_3_) | Gln15 (N_ε2_)  Thr43 (O_ϒ1_) | 2.92 (56.4%)  2.84 (49.6%) | A_(1)_ (O_P2_)  U_(-1)_ (O_2_) | Gln15 (N_ε2_)  Thr43 (O_ϒ1_) | 2.94 (57.2%)  2.81 (56.8%) |
| **hRNase3** |  |  |  | A_(1)_ (N_1_)  A_(1)_ (O)  A_(1)_ (O_P2_)  A_(1)_ (N_6_) | Asn71 (N_δ2_)  His129 (N_δ1_)  Gln15 (N_ε2_)  Asn71 (O_δ1_) | 2.98 (47.9%)  2.83 (47.4%)  2.89 (46.8%)  2.97 (45.1%) |
| **hRNase4** | C_(-1)_ (N_4_)  A_(1)_ (O)  A_(1)_ (O_P1_) | Thr45 (O_ϒ1_)  Phe118 (N)  His117 (N_δ1_) | 2.90 (86.0%)  2.92 (58.0%)  2.78 (49.4%) | U_(-1)_ (N_3_)  U_(-1)_ (O_2_)  A_(1)_ (O)  A_(1)_ (N_1_)  A_(1)_ (N_6_)  A_(1)_ (O) | Thr45 (O_ϒ1_)  Thr45 (N)  Phe118 (N)  Asn71 (N_δ2_)  Asn71 (O_δ1_)  His117 (N_δ1_) | 2.94 (92.6%)  2.92 (88.1%)  2.88 (69.8%)  3.02 (48.7%)  2.96 (48.5%)  2.79 (47.4%) |
| **hRNase5** | C_(-1)_ (N_3_)  C_(-1)_ (O_2_) | Thr44 (O_ϒ1_)  Thr44 (N) | 2.85 (76.3%)  2.90 (70.7%) | U_(-1)_ (O_4_)  U_(-1)_ (N_3_) | Thr44 (N)  Asn43 (O_δ1_) | 2.91 (56.9%)  2.92 (54.2%) |
| **hRNase6** | A_(1)_ (N_6_)  A_(1)_ (N_1_)  A_(1)_ (O)  A_(1)_ (O)  C_(-1)_ (N_4_)  A_(1)_ (N_6_) | Asn69 (O_δ1_)  Asn69 (N_δ2_)  Trp11 (N_ε1_)  Gln15 (N_ε2_)  Thr43 (O_ϒ1_)  Asn65 (O_δ1_) | 2.97 (93.6%)  3.03 (90.4%)  2.87 (87.2%)  2.97 (70.6%)  2.95 (48.2%)  2.97 (47.2%) | A_(1)_ (N_6_)  A_(1)_ (N_1_)  A_(1)_ (N_6_)  A_(1)_ (O) | Asn69 (O_δ1_)  Asn69 (N_δ2_)  Asn65 (O_δ1_)  Gln15 (N_ε2_) | 2.95 (95.6%)  3.05 (85.0%)  2.95 (59.2%)  2.90 (46.7%) |
| **hRNase7** | C_(-1)_ (N_3_)  C_(-1)_ (O_2_)  C_(-1)_ (O_2'_)  C_(-1)_ (N_4_) | Thr43 (O_ϒ1_)  Thr43 (N)  Leu125 (O)  Thr43 (O_ϒ1_) | 2.83 (81.3%)  2.94 (74.8%)  2.71 (60.2%)  3.04 (50.6%) |  |  |  |
