# Supplementary material for: Nucleotide substrate binding characterization in human pancreatic-type ribonucleases
Source: PLoS One. 2019 Aug 8;14(8):e0220037. doi: 10.1371/journal.pone.0220037 (PMC6687278; doi:10.1371/journal.pone.0220037)
Supplement: S4 Table — An interaction is considered as significant if the enzyme-nucleotide pair with interaction energy < -3 kcal/mol of Etotal. Results for central two nucleotides and terminal nucleotides are shown separately. The enzyme residues are listed in the order of decreasing energy of interaction with the nucleotide making the favorable interaction is listed after value. (DOCX) [file pone.0220037.s005.docx]

**Table ST4**

|  | **center nucleotides**  **(ACAC)** | **terminal nucleotides**  **(ACAC)** | **center nucleotides**  **(AUAU)** | **terminal nucleotides**  **(AUAU)** |
| --- | --- | --- | --- | --- |
| **bRNaseA** | His119 (-10.2, A;  -4.0, C)  Thr45 (-4.4, C)  Phe120 (-4.2, C)  Lys66 (-4.1, C)  Val43 (-4.0, C)  Lys7 (-3.9, A)  Asn44 (-3.1, C) | Pro42 (-3.3, A) | His119 (-11.1, A;  -4.0, U)  Phe120 (-5.2, U)  Lys66 (-4.5, U)  Val43 (-3.8, U)  Thr45 (-3.3, U)  Asn71 (-3.1, A) |  |
| **hRNase1** | His119 (-11.5, A;  -4.2, C)  Lys66 (-5.1, C)  Thr45 (-4.5, C)  Phe120 (-4.4, C)  Val43 (-4.4, C)  Arg4 (-4.3, A) | Pro42 (-4.4, A)  Val43 (-3.3, A) | His119 (-11.6, A;  -3.5, U)  Lys66 (-5.3, U)  Val43 (-4.0, U)  Phe120 (-3.9, U)  Lys41 (-3.4, U)  Thr45 (-3.4, U)  Arg4 (-3.4, A)  Asn71 (-3.1, A) | Pro42 (-4.5, A)  Lys7 (-3.1, U)  Val43 (-3.1, A) |
| **hRNase2** | His130 (-8.3, A;  -5.7, C)  Lys39 (-6.1, C;  -4.1, A)  Met1 (-3.3, A)  Gln41 (-3.1, C) | Asn40 (-3.02, C) | His130 (-11.9, A)  Lys39 (-5.5, U;  -4.4, A)  Arg133 (-4.8, U)  Gln41 (-4.4, U) | Arg69 (-4.1, U)  Arg133 (-4.4, A)  Gln41 (-3.2, A) |
| **hRNase3** | His129 (-9.8, A)  Arg35 (-6.6, C)  Arg8 (-5.4, A)  Lys39 (-4.8, C)  Met1 (-4.4, A;  -4.1, C)  Arg2 (-4.0, A) | Met1 (-4.78, C) | His129 (-9.3, A;  -3.7, U)  Lys39 (-4.9, U;  -3.6, A)  Met1 (-3.9, U;  -3.3, A) | Arg2 (-4.4, U)  Arg35 (-3.5, U) |
| **hRNase4** | His117 (-10.0, A;  -4.9, C)  Lys41 (-6.2; C;  -3.5, A)  Phe118 (-5.7, C)  Arg8 (-5.6, A)  Thr45 (-3.2, C)  Lys66 (-3.2, C) | Phe43 (-6.8, A)  Arg8 (-4.4, C) | His117 (-11.8, A;  -4.4, U)  Phe118 (-6.5, U)  Arg8 (-4.3, A)  Lys41 (-4.2, U;  -3.7, A)  Phe43 (-4.2, U)  Thr45 (-3.5, U) | Phe43 (-5.9, A)  Arg8 (-3.9, U) |
| **hRNase5** | His114 (-7.0, A;  -4.2, C)  Lys40 (-6.6, C)  Thr44 (-4.3, C)  Ile42 (-4.0, C)  Leu115 (-3.6, C)  Phe120 (-3.8, C)  Asn43 (-3.5, C) | Arg5 (-4.5, C) | Gln117 (-6.8, U)  His114 (-6.7, A;  -6.5, U)  Arg5 (-4.8, A)  Leu115 (-4.2, U)  Lys40 (-3.6, U)  His13 (-3.5, U)  Asn43 (-3.2, U) | Arg121 (-3.1, A) |
| **hRNase6** | His123 (-9.2, A; -  4.5, C)  Lys39 (-6.6, C)  Lys8 (-6.6, A)  Leu124 (-4.5, C)  Trp11 (-4.1, A)  Thr43 (-3.8, C)  Asn69 (-3.6, A)  Arg67 (-3.4, A)  Gln15 (-3.3, C)  Asn65 (-3.1, A) | Arg5 (-6.4, C)  Gln41 (-3.5, A)  Lys8 (-3.4, C) | His123 (-10.9, A;  -5.3, U)  Leu124 (-5.3, U)  Lys8 (-4.1, A)  Lys64 (-4.0, U)  Asn69 (-3.7, A)  Asn65 (-3.4, A)  Arg67 (-3.4, A)  Thr43 (-3.0, U) | Gln41 (-3.9, A) |
| **hRNase7** | His124 (-9.2, A;  -4.0, C)  Lys39 (-5.5, C)  Thr43 (-4.4, C)  Leu125 (-4.2, C)  Leu41 (-3.5, C)  Asn42 (-3.2, C) | Met1 (-4.6, C)  Leu41 (-4.7, A)  Arg37 (-3.6, C)  Lys2 (-3.9, C) | His124 (-7.3, A;  -3.8, U)  Met1 (-4.7, A)  Lys64 (-3.9, U)  Lys39 (-3.7, U)  Leu125 (-3.2, U) | Trp11 (-4.0,U)  Met1 (-4.0, U)  Leu41 (-3.5, A) |
