# Supplementary figures and images for: Nucleotide substrate binding characterization in human pancreatic-type ribonucleases
Source: PLoS One. 2019 Aug 8;14(8):e0220037. doi: 10.1371/journal.pone.0220037 (PMC6687278; doi:10.1371/journal.pone.0220037)

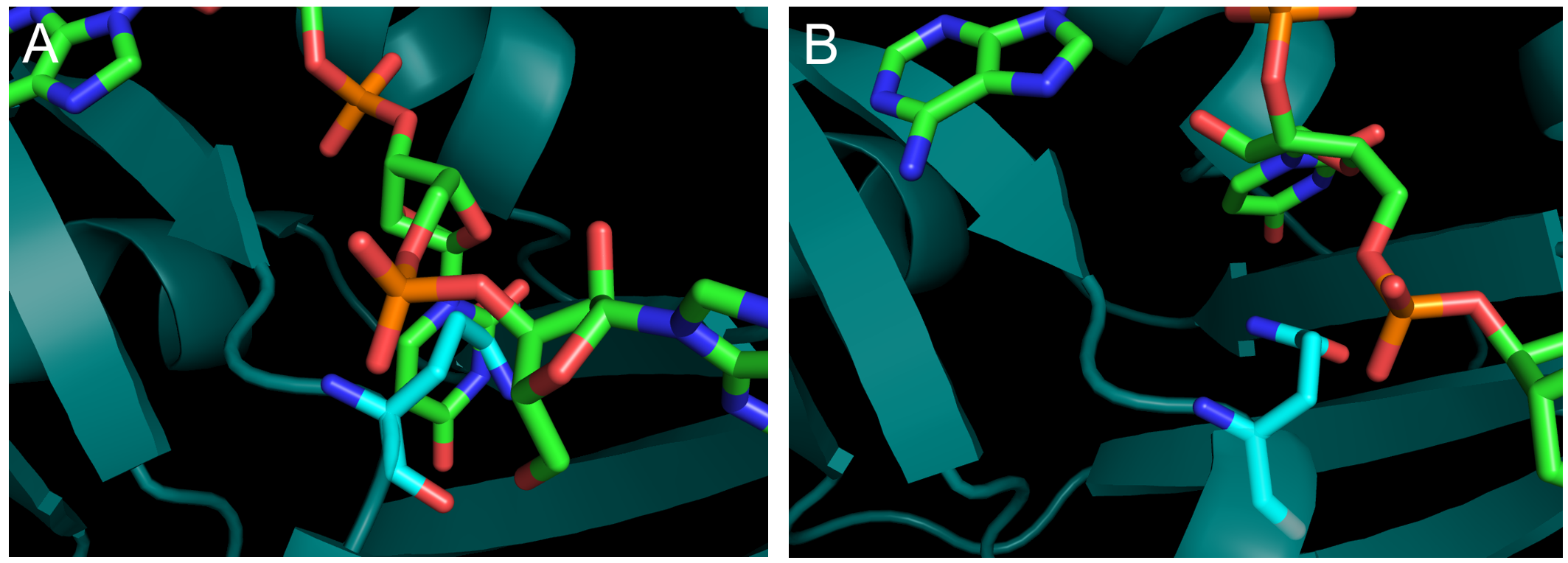

Supplement: S2 Fig — A. Starting conformation, where residue Gln117 has steric clashes with the modeled substrate. B. Ending conformation after the equilibration protocol, where the steric clash between the Gln117 and substrate has been fully resolved. (TIF) [file pone.0220037.s007.tif]

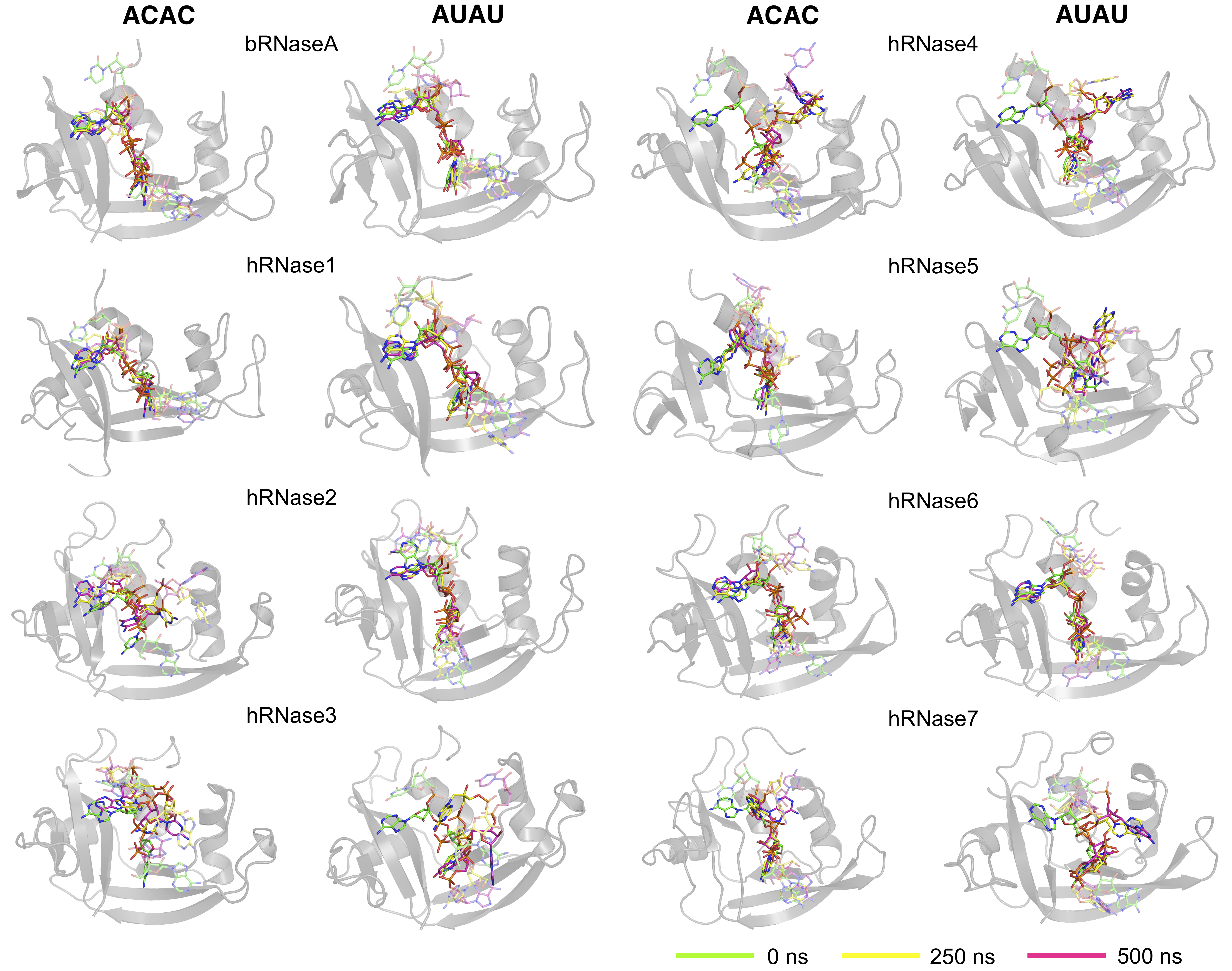

Supplement: S3 Fig — The enzymes are shown as gray cartoons and the 3 relative positions of the substrate at start (0 μs, green), mid-way (0.25 μs, yellow) and end (0.5 μs, magenta) of the simulations are shown as sticks. The results for the two substrates ACAC and AUAU are shown separately. The central two nucleotides of the substrate that interact with two binding sub-sites (B1/P1 or B2/P2) are shown in dark colors, while the two terminal nucleotides (one on each side) are depicted with faded color. The results depicted are from the 310 K simulation trajectories and represent the MD simulations set 1. The results from trajectory 2 at 310 K are qualitatively similar (Table 2). (TIF) [file pone.0220037.s008.tif]

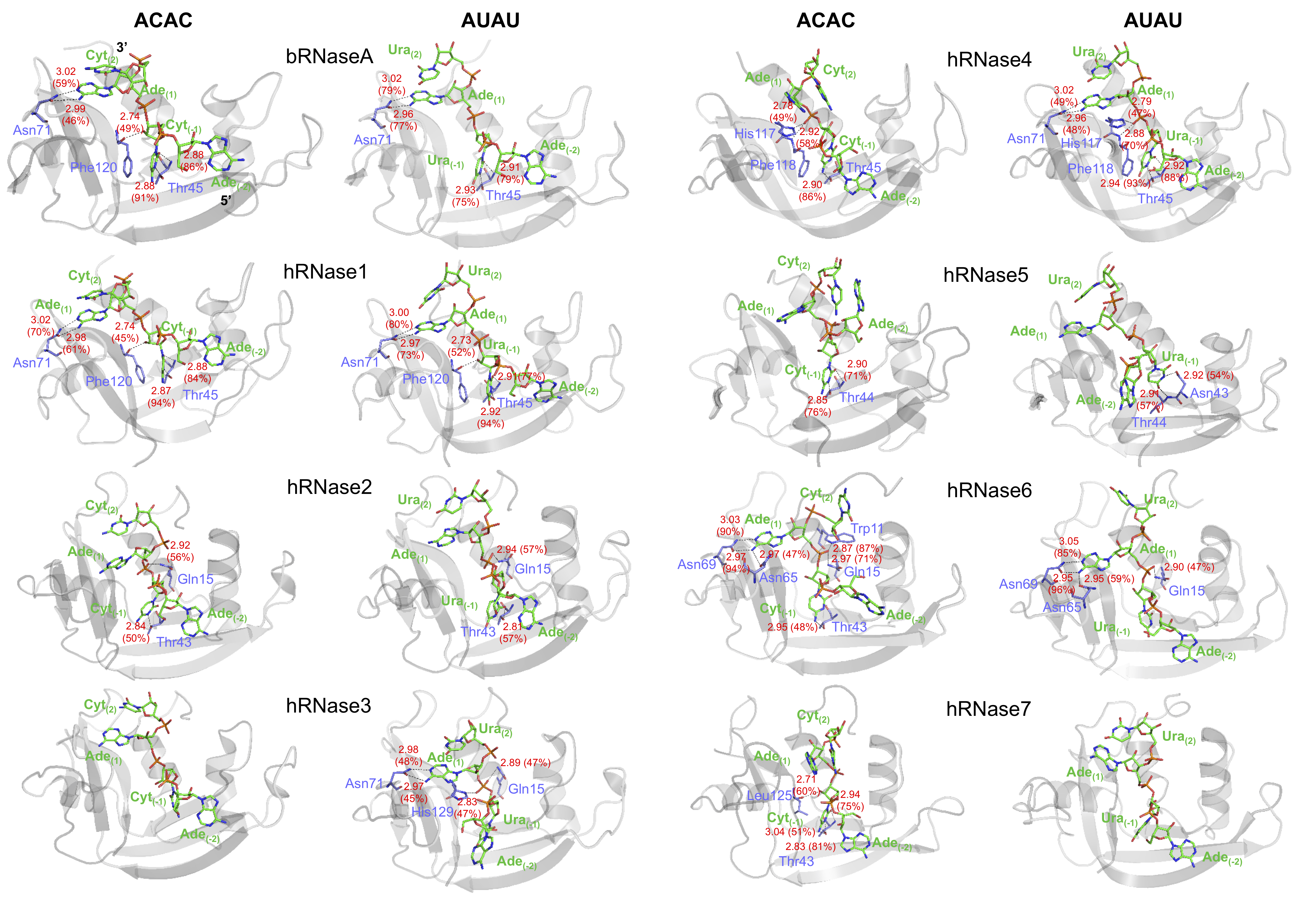

Supplement: S4 Fig — H-bonds (with >45% occupancy) are indicated with black dotted line and averaged bond length (Å) and percentage occupancy are shown in red colored text. Enzyme and nucleotide atoms participating in the H-bonds are labeled, with enzyme residues shown in blue and substrate nucleotides shown in green for carbon atoms, blue for nitrogen, red for oxygen, and orange for phosphorus. Enzyme residue numbers correspond to enzyme sequence, while substrate nucleotides are numbered as A(-2)C/U(-1)A(1)C/U(2), as described in the legend of Fig 1. Note that the substrates orientation (marked by 3’ and 5’ for bRNaseA) appear opposite to the depiction in Fig 1, as enzymes are shown from view used commonly in literature. The percentage occupancies are rounded off to the nearest whole number. (TIF) [file pone.0220037.s009.tif]

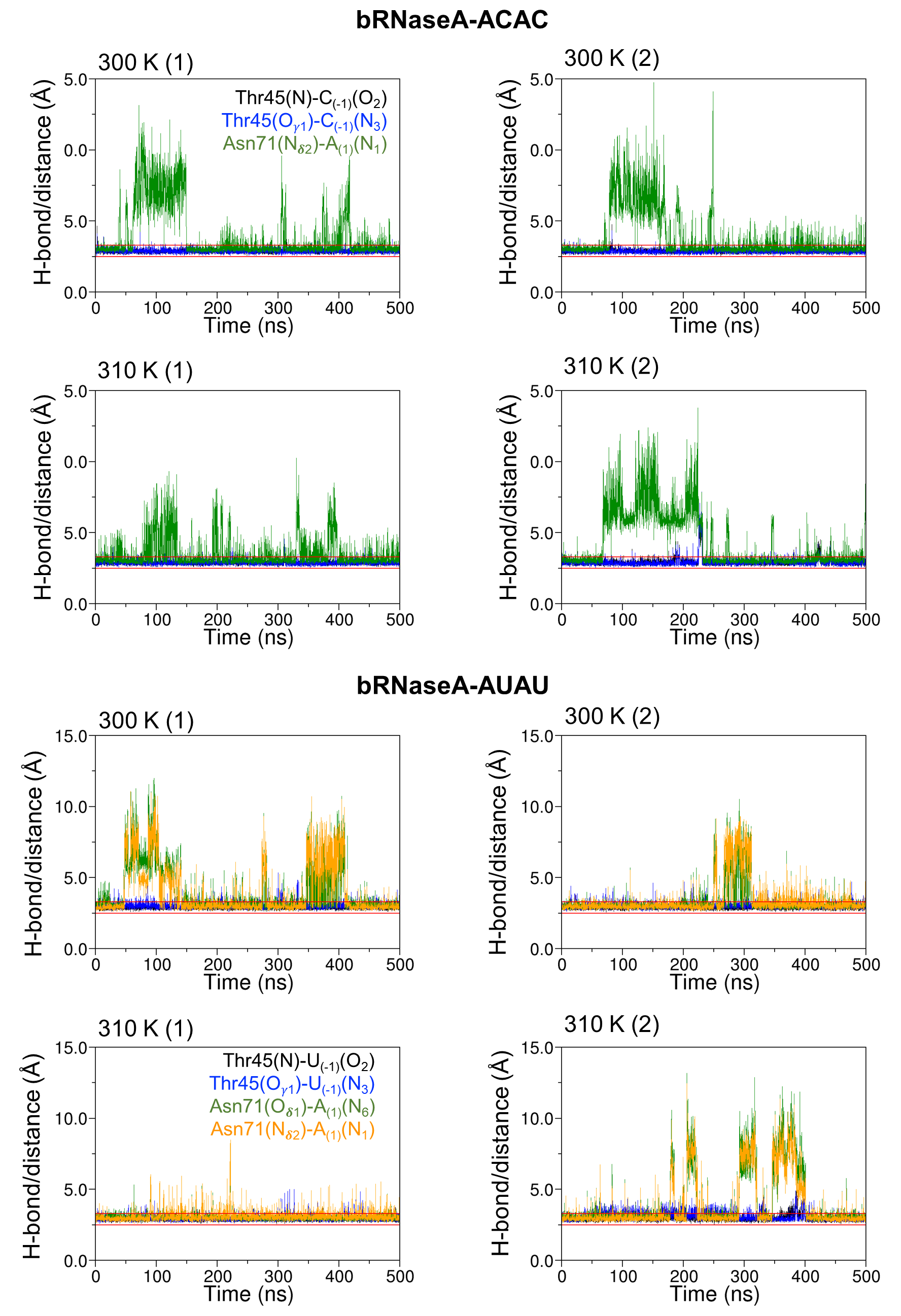

Supplement: S5 Fig — Results from ACAC are shown on top, and from AUAC complex on the bottom. The two alternate trajectories are marked as (1) and (2) for two temperatures 300 K and 310 K. Red lines mark the distance range used for defining H-bonds. (TIF) [file pone.0220037.s010.tif]

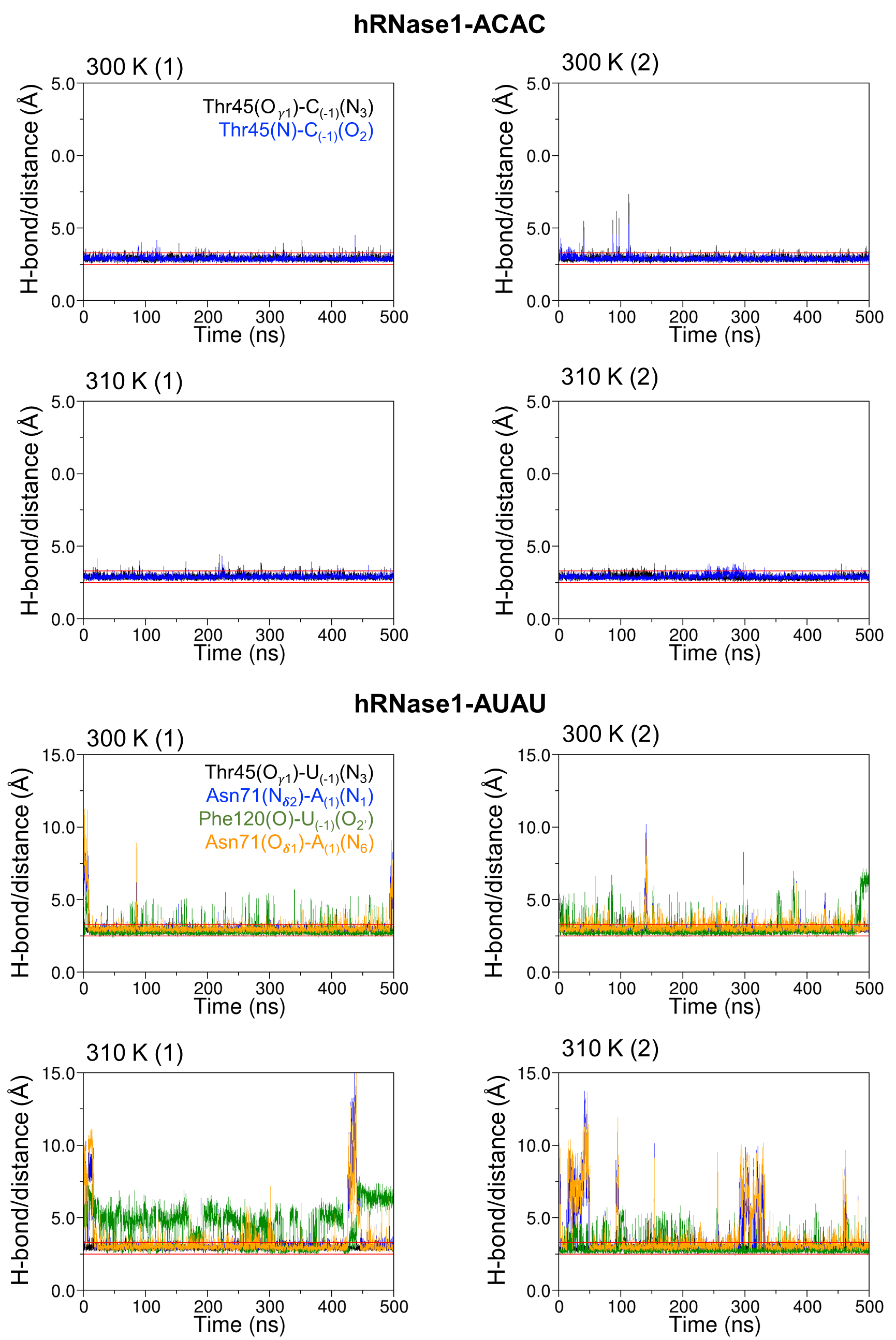

Supplement: S6 Fig — Results from ACAC are shown on top, and from AUAC complex on the bottom. The two alternate trajectories are marked as (1) and (2) for two temperatures 300 K and 310 K. Red lines mark the distance range used for defining H-bonds. (TIF) [file pone.0220037.s011.tif]

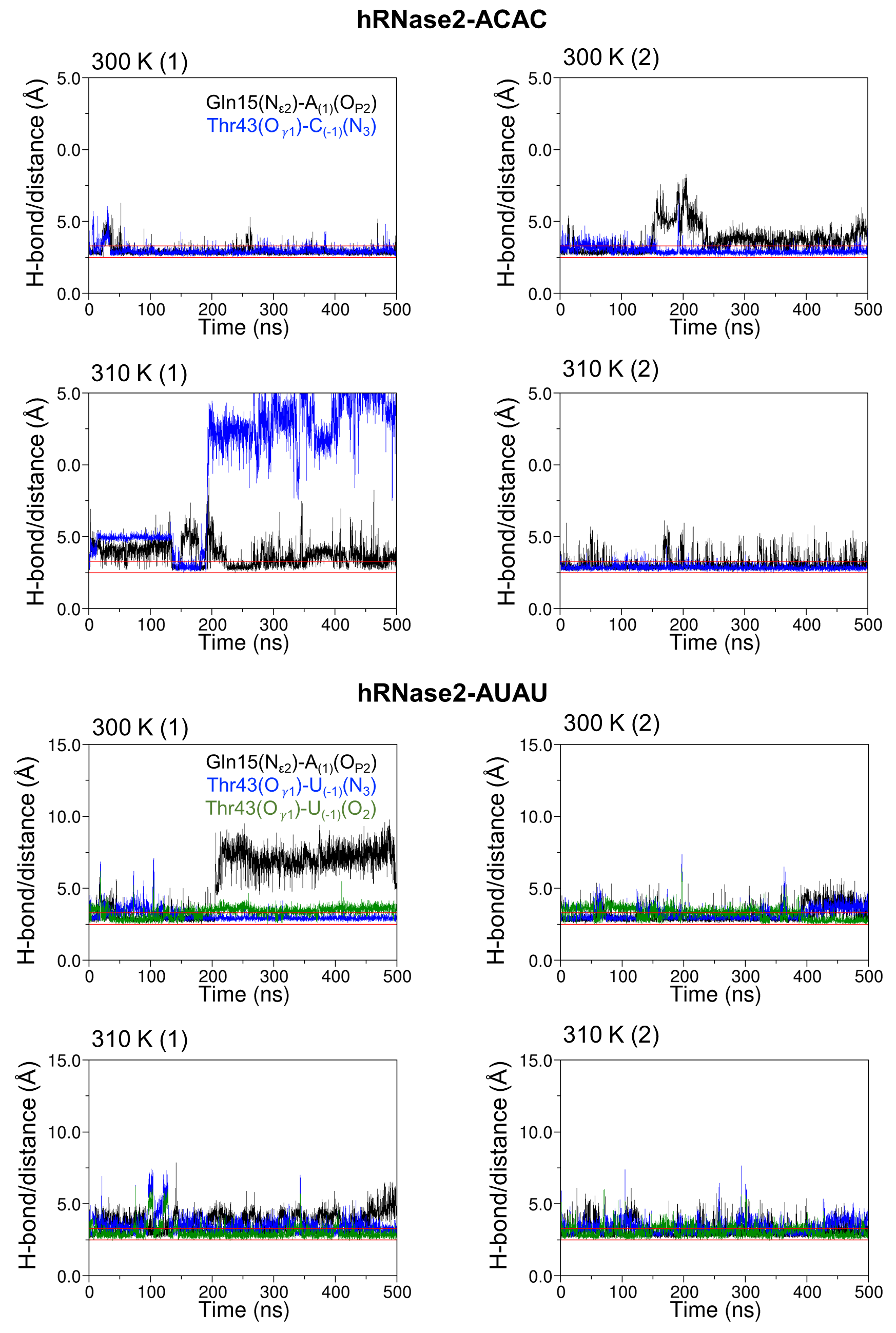

Supplement: S7 Fig — Results from ACAC are shown on top, and from AUAC complex on the bottom. The two alternate trajectories are marked as (1) and (2) for two temperatures 300 K and 310 K. Red lines mark the distance range used for defining H-bonds. (TIF) [file pone.0220037.s012.tif]

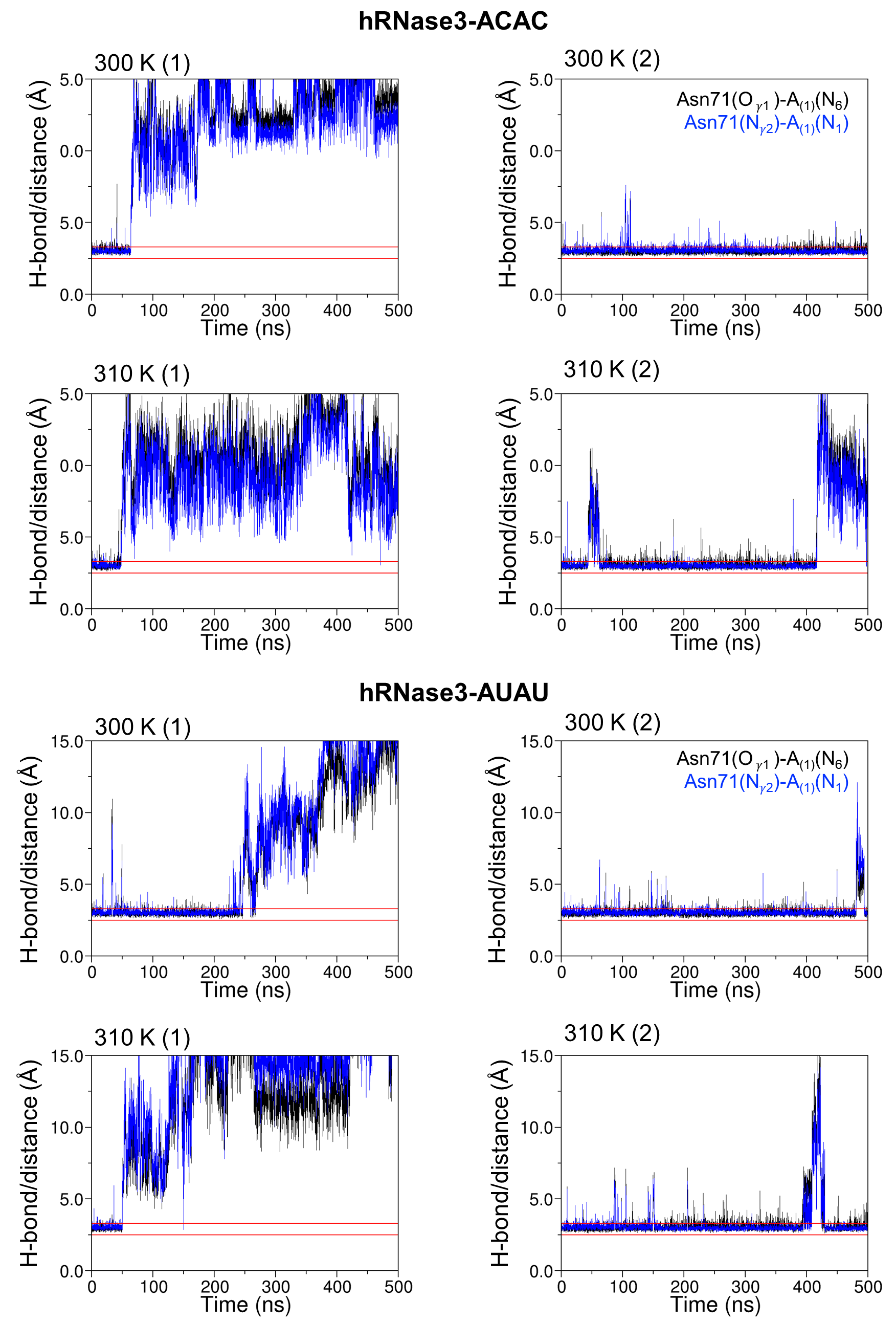

Supplement: S8 Fig — Results from ACAC are shown on top, and from AUAC complex on the bottom. The two alternate trajectories are marked as (1) and (2) for two temperatures 300 K and 310 K. Red lines mark the distance range used for defining H-bonds. (TIF) [file pone.0220037.s013.tif]

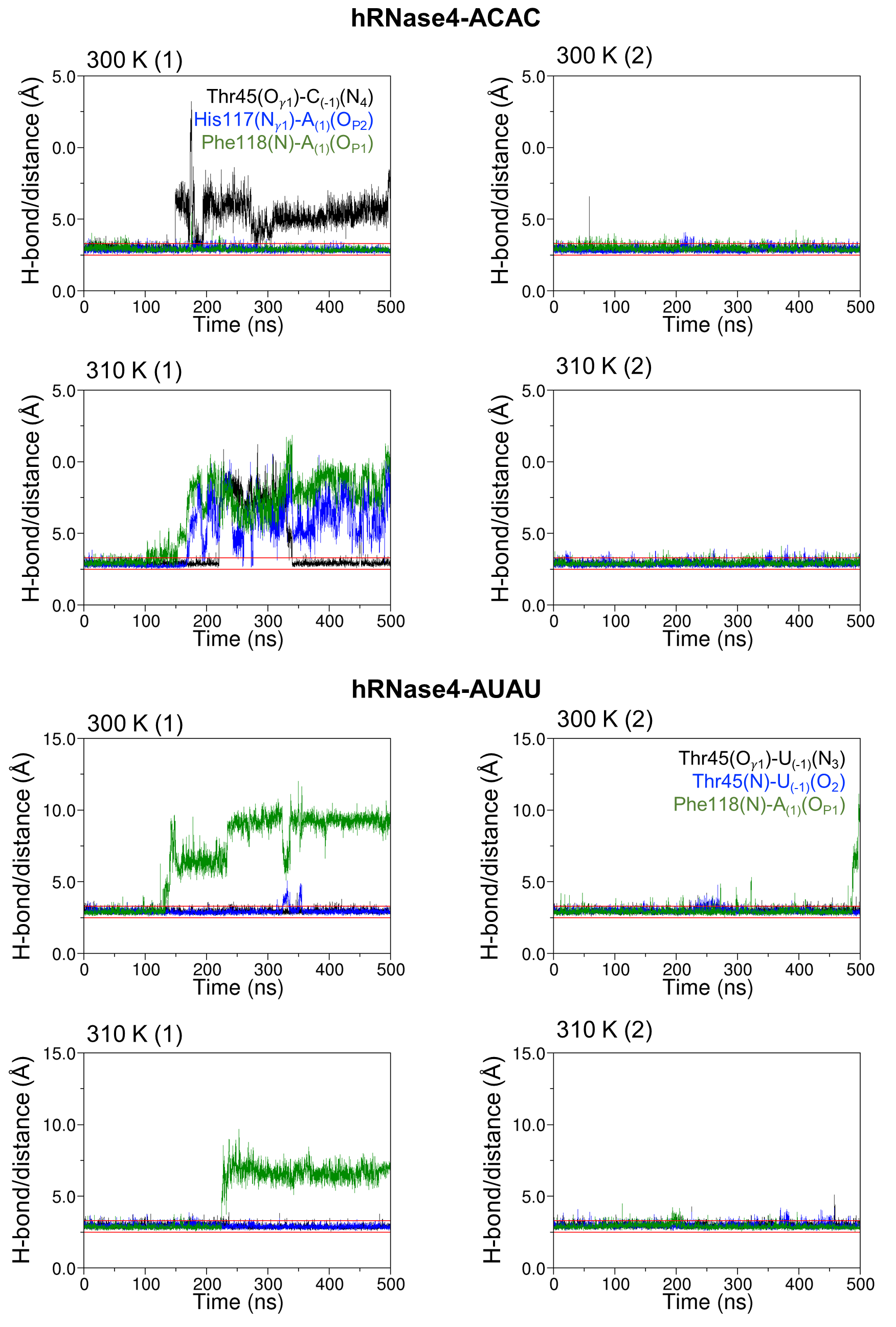

Supplement: S9 Fig — Results from ACAC are shown on top, and from AUAC complex on the bottom. The two alternate trajectories are marked as (1) and (2) for two temperatures 300 K and 310 K. Red lines mark the distance range used for defining H-bonds. (TIF) [file pone.0220037.s014.tif]

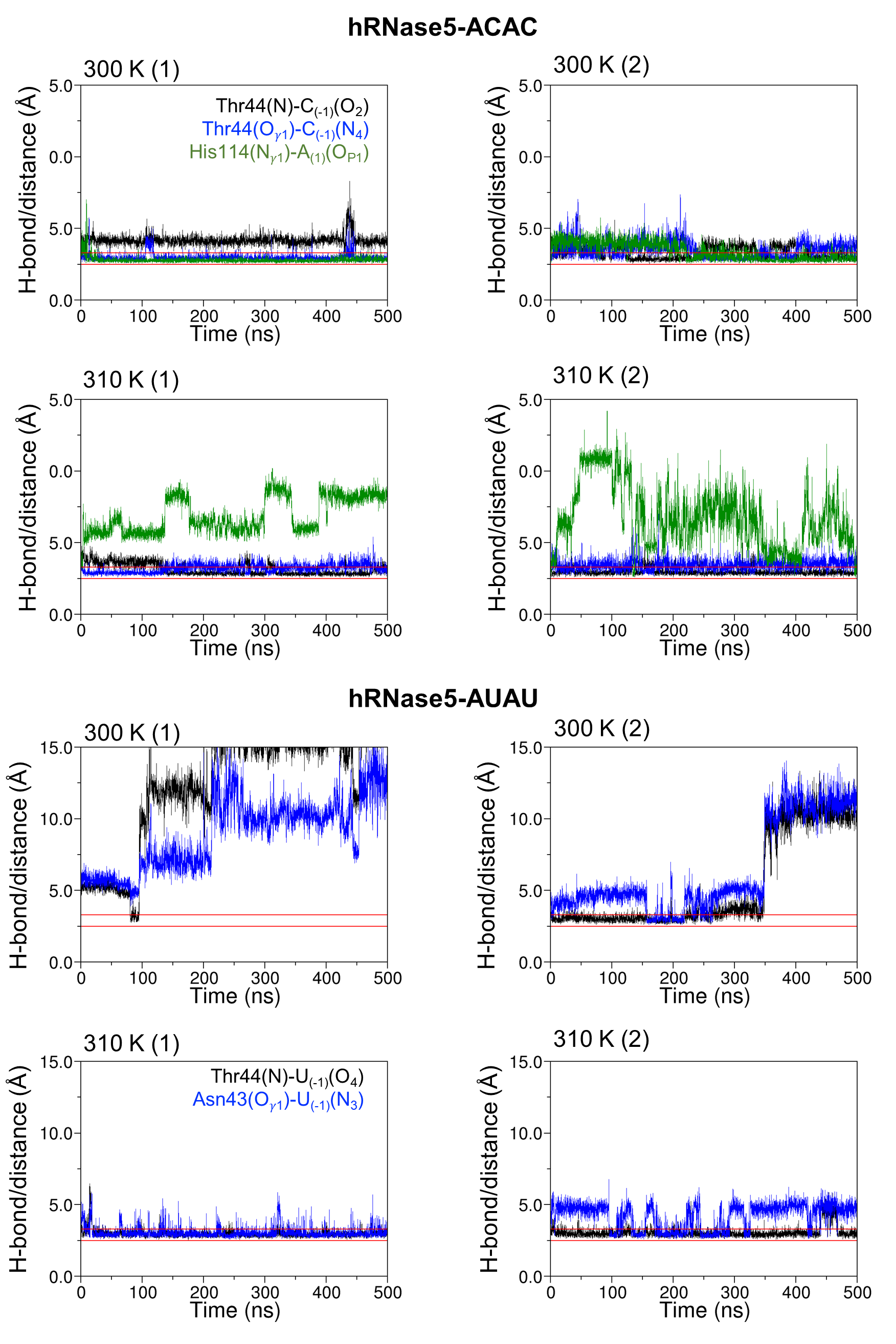

Supplement: S10 Fig — Results from ACAC are shown on top, and from AUAC complex on the bottom. The two alternate trajectories are marked as (1) and (2) for two temperatures 300 K and 310 K. Red lines mark the distance range used for defining H-bonds. (TIF) [file pone.0220037.s015.tif]

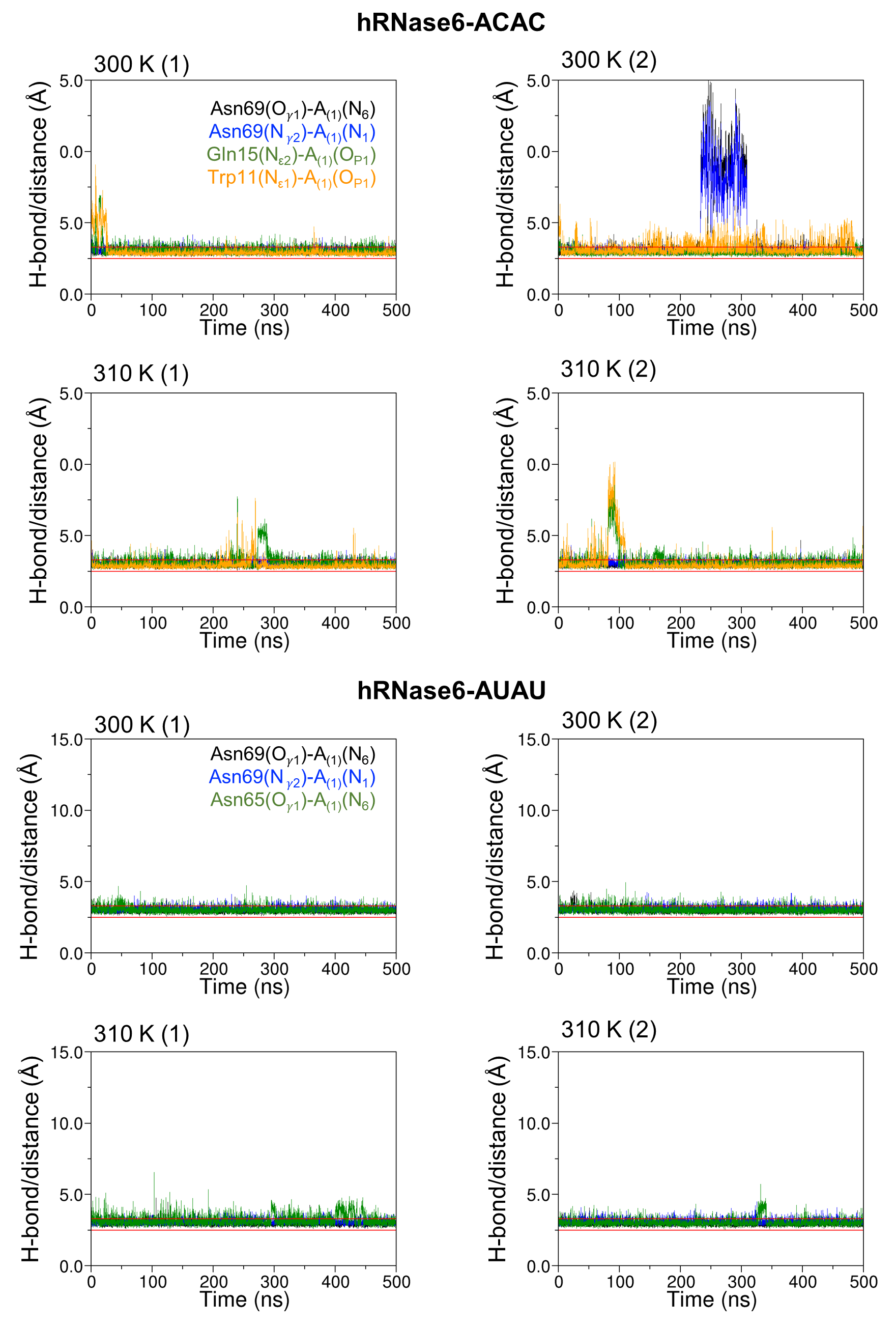

Supplement: S11 Fig — Results from ACAC are shown on top, and from AUAC complex on the bottom. The two alternate trajectories are marked as (1) and (2) for two temperatures 300 K and 310 K. Red lines mark the distance range used for defining H-bonds. (TIF) [file pone.0220037.s016.tif]

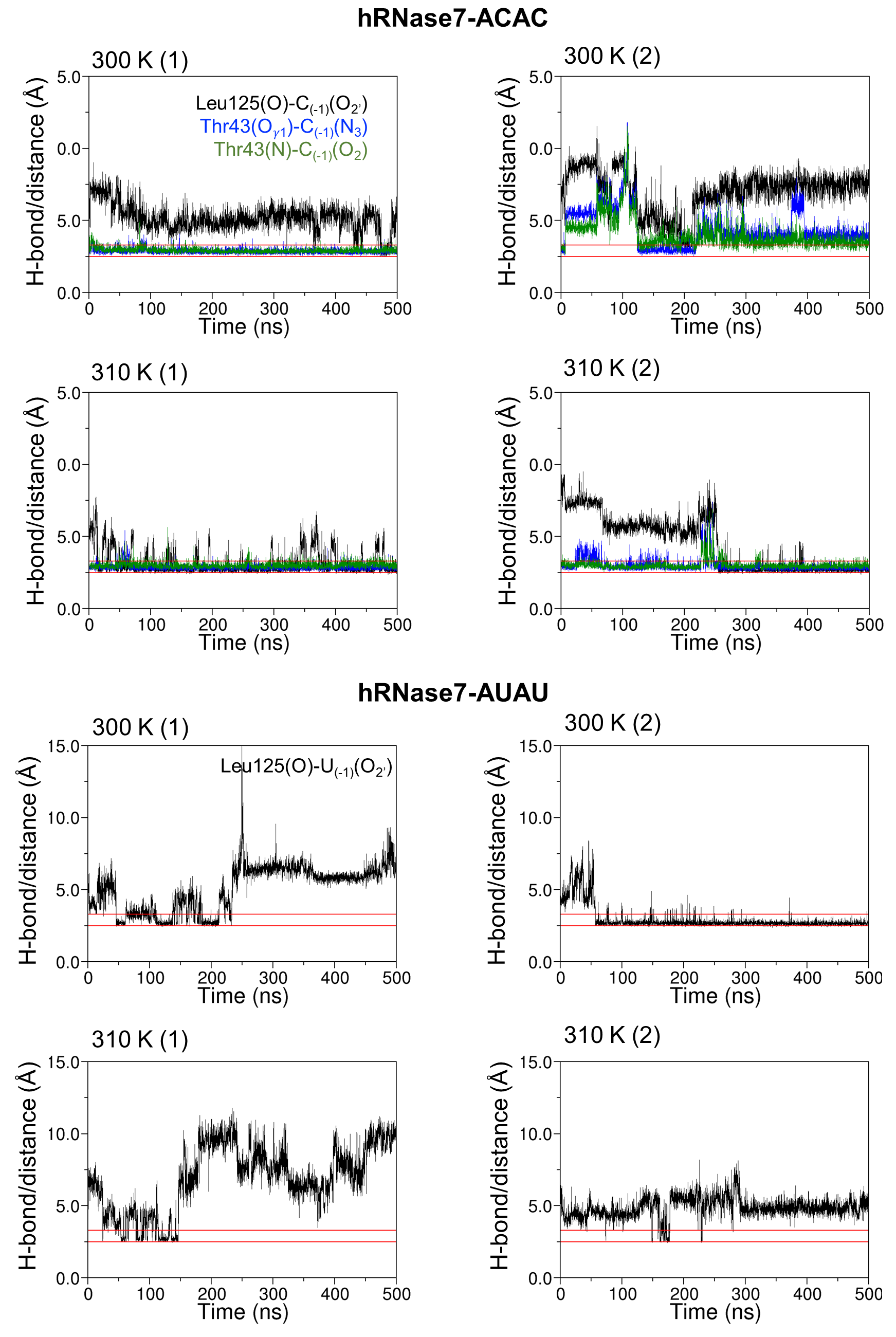

Supplement: S12 Fig — Results from ACAC are shown on top, and from AUAC complex on the bottom. The two alternate trajectories are marked as (1) and (2) for two temperatures 300 K and 310 K. Red lines mark the distance range used for defining H-bonds. (TIF) [file pone.0220037.s017.tif]

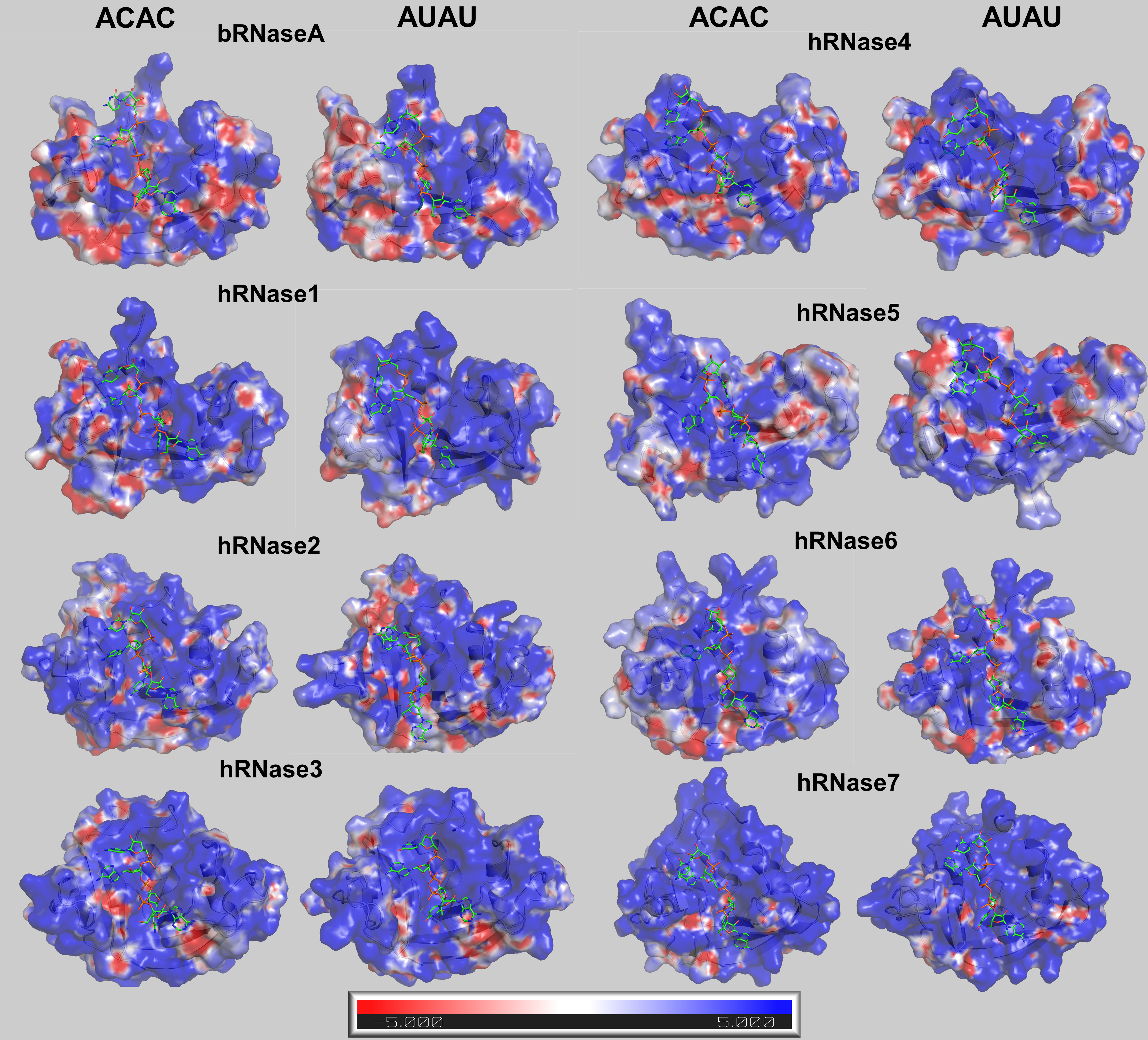

Supplement: S13 Fig — Enzyme conformation from the MD ensemble closest to the averaged structure for the entire trajectory (conformation with smallest RMSD with averaged structure) was used to calculate the representative electrostatic potential (+5kT/e in blue and –5kT/e per electron in red). (TIF) [file pone.0220037.s018.tif]

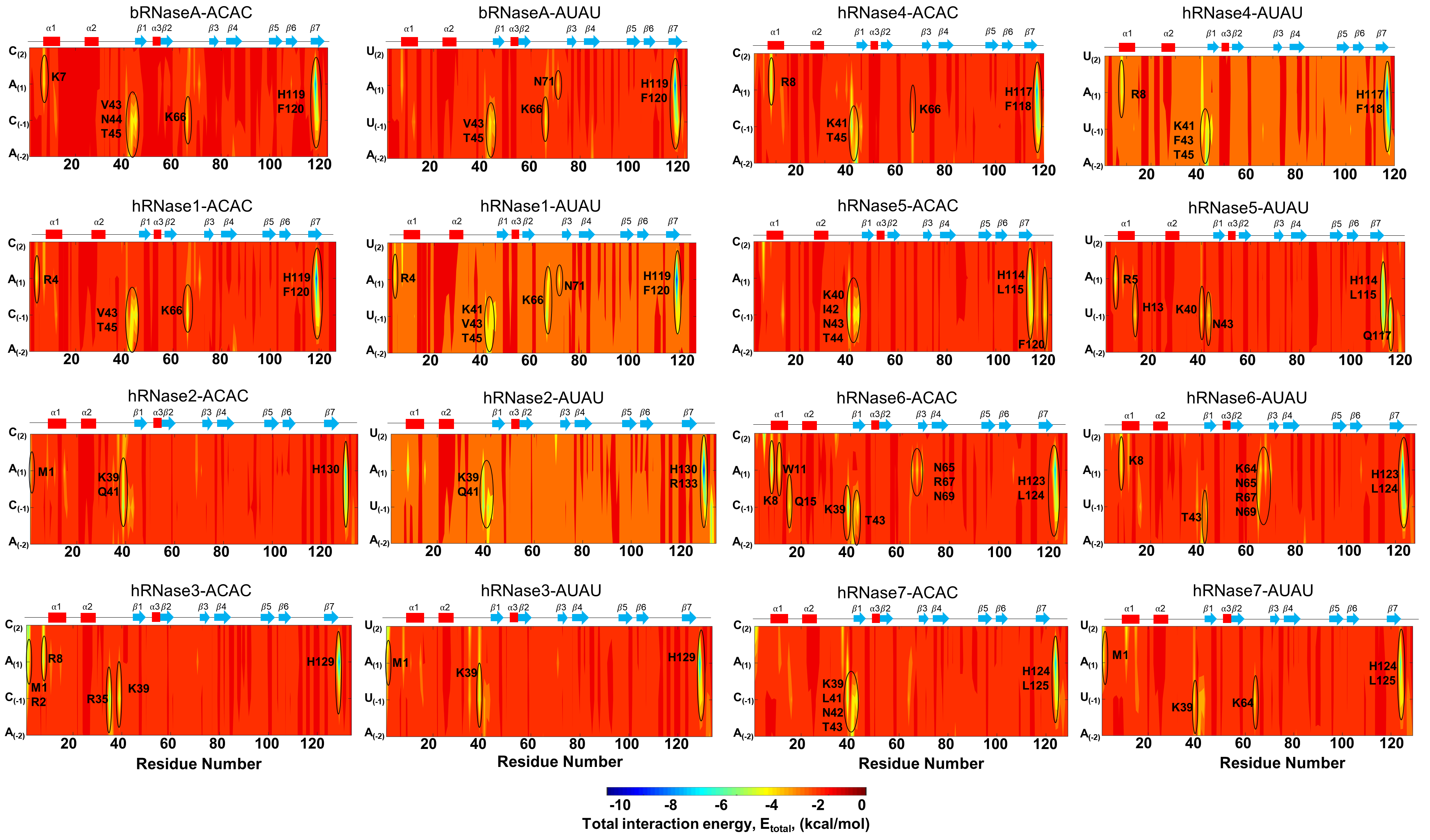

Supplement: S14 Fig — Total interaction energy was computed as a summation of electrostatic and van der Waals interaction energy between atom pairs of enzyme residue and substrate nucleotide (ACAC and AUAU, respectively). The areas of largest negative energy (blue) represent favorable interactions, while positive energy (red) represents less favorable or unfavorable interactions. For ease of comparison, sequence of the model substrate has been adjusted to match the orientation depicted in Figs 2 and 3 (CACA and UAUA). Enzyme residues showing total interaction energy (Etotal, S3 Table) contributions < -3 kcal/mol were considered significant and are marked with ellipses. (TIF) [file pone.0220037.s019.tif]
